# MED12 mutation induces RTK inhibitor resistance in NSCLC via MEK/ERK pathway activation by inflammatory cytokines

**DOI:** 10.1007/s00018-025-05791-w

**Published:** 2025-08-20

**Authors:** Hyun-Min Ryu, Deokhoon Kim, Jun Young Choi, Shinkyo Yoon, Ho-Su Lee, Ji Eun Park, Eunjin Lee, Yunkyung Sung, Chang Hoon Lee, Eun-Young Lee, Wanlim Kim, Seyoung Seo, Sang-We Kim, Kang-Seo Park, Dae Ho Lee

**Affiliations:** 1 Department of Oncology, Asan Medical Center, University of Ulsan, College of Medicine, 88 Olympic-ro 43-gil, Songpa-gu, Seoul, 05505 Republic of Korea; 2 Department of Biomedical Sciences, Asan Medical Center, University of Ulsan, College of Medicine, 88 Olympic-ro 43-gil, Songpa-gu, Seoul, 05505 Republic of Korea; 3 Department of Pathology, Asan Medical Center, University of Ulsan College of Medicine, Seoul, 05505 Republic of Korea; 4 Department of Biochemistry and Molecular Biology, University of Ulsan College of Medicine, Seoul, Republic of Korea; 5 Department of Radiology and Research Institute of Radiology, College of Medicine, University of Ulsan, Asan Medical Center, Seoul, Republic of Korea; 6 SCBIO Inc, Daejeon, 34141 Republic of Korea; 7 Department of Biochemistry College of medicine, Soonchunhyang University, Cheonan, Republic of Korea; 8 Department of Orthopaedic Surgery, Asan Medical Center, University of Ulsan College of Medicine, Seoul, 05505 Republic of Korea

**Keywords:** NSCLC, MED12 mutation, Drug resistance, Receptor tyrosine kinase inhibitor, MEK inhibitor

## Abstract

Non-small cell lung cancer (NSCLC) is frequently associated with mutations in receptor tyrosine kinases (RTKs), such as EGFR and ALK. While RTK inhibitors (RTKIs) have proven effective in treating patients with specific RTK mutations, the emergence of resistance to these therapies remains a significant clinical obstacle. As such, there is still an unmet need for the identification of new biomarkers that can predict resistance to RTK inhibitors in clinical use. In the present study, we demonstrate that MED12 mutations are a key driver of RTKi resistance in NSCLC cells. This resistance is mediated through the release of inflammatory cytokines triggered by MED12 degradation. Notably, we observed that of the two major downstream signaling pathways activated by inflammatory cytokines, only the MEK/ERK pathway was upregulated, while the PI3K/AKT pathway was unaffected in MED12 knock-out (KO) cells. The degradation of MED12 results in the dissociation of the MED12 complex, which subsequently leads to YAP phosphorylation. This phosphorylated YAP increases PTEN expression by inhibiting miR-29, thereby suppressing the PI3K/AKT signaling pathway. Importantly, treatment with trametinib, a MEK inhibitor, effectively suppressed tumor growth in MED12KO NSCLC cells and in xenograft models derived from these cells. These findings suggest that targeting the MEK/ERK signaling pathway, such as with trametinib, may provide a viable strategy to overcome RTKi resistance in MED12-mutant NSCLC. Furthermore, MED12 is identified as a crucial biomarker and potential therapeutic target for overcoming RTKi resistance.

**Supplementary Information:**

The online version contains supplementary material available at 10.1007/s00018-025-05791-w.

## Introduction

Molecular targeted therapy has changed the cancer treatment paradigm, with many targeted agents, including ALK tyrosine kinase inhibitor (ALK-TKI) and EGFR tyrosine kinase inhibitor (EGFR-TKI) now standard therapies for patients harboring a corresponding molecular target. However, although many patients benefit from this therapy, most eventually succumb to their disease due to the occurrence of resistance. Anaplastic lymphoma kinase (ALK) gene rearrangement is often observed in non-small cell lung cancer (NSCLC), accounting for 3–5% of patnts with lung adenocarcinoma [1–4]. Ceritinib, a second-generation ALK-TKI, significantly prolongs progression-free survival (PFS) with only moderate adverse events compared with crizotinib, a first-generation ALK-TKI. Second-generation ALK-TKIs, including alectinib, brigatinib, and ceritinib, are the standard first-line therapy for patients with ALK-rearranged NSCLC [5–8]. A third-generation ALK-TKI, lorlatinib, has been introduced as a first and second-line treatment option [9]. However, many patients still show resistance to ALK-TKIs, although they benefit from the higher efficacy of ALK-TKIs compared with other receptor tyrosine kinase inhibitors (RTKi) [10].

Several mechanisms contribute to ALK inhibitor resistance, including secondary ALK mutations and recursive another RTK activation. The secondary or resistance mutation in the ALK domain (i.e., G1202R and I1171T/N/S) decreases the TKI-binding affinity for ALK and its efficacy, explaining the occurrence of acquired resistance to second-generation ALK inhibitors in less than 30–40% of patients [11–15]. Tumors can activate other RTK signaling pathways, bypass ALK downstream pathways, and induce resistance. They can also amplify the ALK gene, leading to increased expression of the ALK protein and reduced effectiveness of ALK inhibitors [16–19]. Epithelial-to-mesenchymal transition (EMT) is a process in which cancer becomes more invasive and resistant to treatment [20–23]; however, various mechanisms remain elusive, including those involved in activating the RTK bypass pathway. Researchers are exploring several strategies to overcome ALK inhibitor resistance, including developing a new ALK, combining ALK inhibitors with other targeted therapies, chemotherapy, or immunotherapy to boost the ability of the immune system to attack tumors [10]. However, resistance to ALK inhibitors is complicated, complex, and multifaceted.

Mediator complex subunit 12 (MED12) is a key component of the transcriptional MEDIATOR complex [24–27], and essential in transcriptional regulation via RNA polymerase II. Several studies have highlighted the important role of MED12 in human tumors, mutations that frequently occur in uterine leiomyomas, breast tumors, and prostate adenocarcinoma [28–31]. MED12, a protein originally associated with transcriptional regulation, is critical in drug response modulation in various types of cancer. Ten years ago, Huang et al. conducted a large-scale RNAi screen to identify potential predictors of response to ALK and EGFR inhibitors in NSCLC cells. Their study highlighted MED12 as a crucial determinant of drug response and interestingly a fraction of MED12 localizes in the cytoplasm, wherein it physically interacts with TGFβR2, a component of the TGF-β receptor signaling pathway. In addition, MED12 has been reported to negatively regulate TGFβR2 signaling. In MED12-deficient cells, the inhibition of TGF-βR signaling restored drug responsiveness, indicating that MED12 acts upstream of TGF-βR signaling, and its suppression leads to resistance to EGFR, ALK, and BRAF inhibitors [32]. Thus, targeting MED12 could be a potential strategy to combat drug resistance in these NSCLC subtypes. Rosell et al. corroborated the significance of MED12 deficiency in resistance to tyrosine kinase inhibitors in EGFR-mutant NSCLC [33]. This observation reinforces that MED12 is pivotal in modulating drug response in lung cancer and underscores its potential as a biomarker for predicting therapeutic outcomes.

In contrast to NSCLC, the function of MED12 appears intact in small-cell lung cancer [34], but assumes a different role in mediating drug response in breast cancer. A study by Lu Wang et al. demonstrated that higher levels of MED12 and CARM1 predict better response to chemotherapeutics in breast cancer [35]. CARM1-mediated methylation of MED12 sensitizes breast cancer cells to 5-fluorouracil but not to RTK inhibitors, suggesting a unique mechanism of drug response modulation in breast cancer compared with NSCLC. Additionally, MED12 methylation was associated with elevated levels of p21/WAF1, which is associated with a poor prognosis in breast cancer patients undergoing chemotherapy. Thus, MED12 methylation could serve as a predictive biomarker for these patients [35].

Collectively, these studies demonstrate that MED12 alterations, both transcriptionally and post-translationally, play a dual role in modulating chemotherapeutic response in various forms of cancer. Targeting the MED12 signaling pathway holds promise as a novel strategy to combat drug-resistant cancers, and understanding the specific roles of MED12 in different cancer subtypes is crucial for developing personalized treatment approaches and improving patient outcomes. Although a paper published ten years ago reported that MED12 mutations induce RTKi resistance [32], the exact mechanism of resistance caused by MED12 suppression remains still unclear and this previous study has not specifically presented a clinical treatment strategy that can be directly applied to overcome RTK inhibitor resistance caused by the decreased expression of MED12.

In this study, we also found that MED12 mutation induces RTKi resistance via MED12 degradation in NSCLC cells. However, our MED12 mutation-induced RTKi resistant cell lines did not exhibit the previously known MED12-related resistance mechanisms mentioned above.

This study aims to provide new insights into the RTKi resistance mechanisms caused by MED12 mutations in NSCLC, propose immediately applicable treatment strategies to overcome this resistance, and highlight the importance of MED12 as a companion diagnostic marker and therapeutic target.

## Materials and methods

### Cell culture and reagents

H3122 and PC9 cell lines were purchased from the American Type Culture Collection (Manassas, VA) and cultured in RPMI 1640 (cat. LM011-01, WELGENE), supplemented with 10% fetal bovine serum (cat. S101-07, WELGENE), 1 mM sodium pyruvate (cat. LS013-01, WELGENE), 10 mM HEPES (cat. BB001-01, WELGENE) and 100 IU/mL Penicillin-Streptomycin Solutions (cat. LS202-02, WELGENE) at 37℃, 5% CO2. Trametinib (cat. S2673), ceritinib (cat. S7083), alectinib (cat. S2762), lorlatinib (cat. S7083), osimertinib (cat. S7297) and Z-VAD-FMK (cat. S7023)were purchased from Selleck Chemicals LLC (Houston, TX). All drugs were dissolved in DMSO in the in vitro studies. Ceritinib and trametinib were formulated in 0.5% methylcellulose/0.5% Tween 80 (vehicle) in the in vivo studies.

### Establishing the ceritinib-resistant (CR) and MED12 knockout (KO) cell line

To establish the H3122CR cell line, the H3122 cell line was cultured in complete media with a gradual increase in the concentration of ceritinib. The concentration was initiated at 3 nM and gradually escalated over approximately 3 months until 1 µM. Fresh media containing ceritinib was replaced every 2–3 days.

The MED12 KO cell line was generated using the CRISPR/Cas9 system. The pLentiCRISPR v2 plasmids containing both Cas9 and MED12 gRNA (GGATCTTGAGCTACGAACA C, GGGGTCCTGAGGGTAAACAT) were purchased from GenScript. These plasmids and psPAX2 (plasmid #12260, Addgene) and pMD2.G (plasmid #12259, Addgene) were transfected into HEK293T cells for virus packaging. The H3122 and PC9 cell lines were seeded in a 6-well plate and infected with the lentivirus soup. Selection was performed by treating the cells with 1 µg/mL puromycin for 2 weeks. Additionally, single clone selection was performed by seeding 0.5 cells per well in a 96-well plate.

### Targeted sequencing and analysis

The Agilent SureSelect Target Enrichment protocol for the Illumina paired-end sequencing library (Version C2, December 2018) with 1ug input gDNA to generate standard exome capture libraries was utilized. The SureSelect Human All Exon V5 probe set was used in all cases. PicoGreen and agarose gel electrophoresis measured the quantification and quality of DNA. 1 µg of genomic DNA diluted in EB Buffer from each cell line was sheared to a target peak size of 150–200 bp using the Covaris LE220 focused-ultrasonicator (Covaris, Woburn, MA, USA) according to the manufacturer’s instructions. The 8 microTUBE strip was loaded into the tube holder of the ultrasonicator, and the DNA was sheared using the following settings: mode, frequency sweeping; duty cycle, 10%; intensity, 5; cycles per burst, 200; duration, 60 Sect. 6 cycles; temperature, 4–7 °C. The fragmented DNA was repaired by ligation of ‘A’ to the 3′ end, and agilent adapters were ligated to the fragments. After assessing the ligation, the adapter-ligated product was PCR amplified. For exome capture, 250 ng of DNA library was mixed with hybridization buffers, blocking mixes, RNase block, and 5 µL of SureSelect all exon capture library, according to the standard Agilent SureSelect Target Enrichment protocol. Hybridization to the capture baits was conducted at 65 °C using a heated thermal cycler lid option at 105 °C for 24 h on a PCR machine. The captured DNA was then washed and amplified. The final purified product was then quantified using qPCR according to the qPCR Quantification Protocol Guide (KAPA Library Quantification kits for Illumina Sequencing platforms) and qualified using the TapeStation DNA screen tape D1000 (Agilent, California, USA). Sequencing was then performed using the HiSeq™ 2500 platform (Illumina, San Diego, USA).

The sequence mapping steps were performed per a method described elsewhere [36]. Somatic variant calling for single nucleotide variants and short indels was conducted using VarDict [37]. Germline variants of candidates for somatic variants (found in > = 1% of samples) were filtered out with a common germline variants database (dbSNP [build 141], gnomAD, common germline variants from 1100 healthy Koreans) [38].

### Recovery of wild-type MED12 and mutant MED12 expression in the MED12 KO cell line

MED12 (NM_005120) Human Tagged ORF Clone (CAT#: RC217206) was purchased from OriGene Technologies. Bioneer Corporation was then commissioned to generate the MED12 L1283P (3848T > C) mutation and GFP-tag (C-terminal) in the plasmid.

The H3122 cell line was seeded in a 60 mm cell culture dish and transfected with wild-type MED12 and mutant MED12 plasmid using Lipofectamine™ 3000 Transfection Reagent (cat. L3000015, Invitrogen™). The cells were treated with 1 µg/mL puromycin for approximately 2 weeks for antibiotic selection. To induce expression of wild-type MED12 in the clones, single colonies showing strong GFP expression were selected using cloning cylinders. The clones were numbered #1, #2, and #3 in the order of increasing GFP expression. The clones expressing the mutant MED12 were chosen by random selection of single colonies, regardless of GFP expression strength.

### Overexpression of various YAP overexpression in the parental and MED12 KO cell lines

EFSp-GFP-Empty (Plasmid #174171), EFSp-GFP-YAP (Plasmid #174168), EFSp-GFP-YAP (5SA, Plasmid #174170) and EFSp-GFP-YAP (S94A, Plasmid #174169) [39] were purchased from Addgene. Virus packaging was performed by transfecting HEK293T cells with these plasmids and psPAX2 (plasmid #12260, Addgene) and pMD2.G (plasmid #12259, Addgene). The H3122 and H3122/MED12 KO cell lines were cultured in a 6-well plate and exposed to a lentivirus-containing medium. Following infection, the cells were subjected to puromycin selection at a concentration of 1 µg/mL for 2 weeks.

### Cell viability assay

The cells cultured in a cell culture dish were detached using TrypLE™ Express Enzyme (cat. 12604021, Gibco™), then cell counting was performed. The cells were seeded in a 96-well plate at a density of 2–3 × 10^3 cells/well with 100 µL of medium. After overnight incubation, the cells were treated with drugs at a maximum concentration of 1 µM using a half-dilution scheme and incubated at 5% CO2 and 37 °C. After 72 h, CellTiter 96^®^ AQueous One Solution (MTS solution, G3581) purchased from Promega Corporation was added at a volume of 20 µL per well. After incubating for 1–4 h, the absorbance at 490 nm was measured using a microplate spectrophotometer.

### Caspase 3/7 activity assay

Caspase 3/7 activity was measured using the Caspase-Glo^®^ 3/7 Assay Kit (Promega, Cat# G8090) following the manufacturer’s instructions. Cells were seeded in white 96-well plates at a density of 2–3 × 10^3^ cells/well and incubated overnight. The next day, cells were treated with ceritinib, osimertinib, or trametinib at six different concentrations, ranging from 1000 nM to 32 nM (2-fold serial dilutions), for 72 h. Z-VAD-FMK was used at a concentration of 20 µM.

After treatment, an equal volume of Caspase-Glo^®^ reagent was added to each well containing culture medium, followed by incubation at room temperature for 1 h in the dark. Luminescence was then measured using a microplate reader.

To ensure accuracy, caspase activity values were normalized to cell viability data obtained from parallel MTS assays performed under the same conditions. Caspase activity values were normalized to cell viability and are presented as fold change relative to the control condition.

### Western blotting analysis

The cells were lysed using a lysis buffer supplemented with protease inhibitors, phosphatase inhibitors, and EDTA. The lysates were quantified to reach a concentration of 2 µg/µL using the BCA assay. The proteins present in the lysates were separated by SDS-PAGE gel and transferred onto PVDF membranes. The primary antibody was purchased from Cell Signaling Technology, and the following antibodies were used: MED12 (#14360), β-actin (#8457), phospho-AKT (#4060), phospho-ERK1/2 (#9101), phospho-ALK (#3341), cleaved PARP (#5625), CDK8 (#17395), MED13(#91684), CCNC (#68179), YAP (#14074), phospho-YAP (#13008), PTEN (#9559), and ubiquitin (#3933). The following secondary antibodies were used: Goat anti-Rabbit IgG (H + L)-HRP Conjugate (cat. 1706515) and Goat anti-Mouse IgG (H + L)-HRP Conjugate (cat. 1706516).

### TaqMan MicroRNA assay

Total RNA was extracted from cells using the miRNeasy Mini Kit (cat. 217004, Qiagen). 10 ng of total RNA, as measured by the NanoDrop™ 2000/2000c Spectrophotometer, was reverse transcribed into cDNA using the TaqMan™ MicroRNA Reverse Transcription Kit (cat. 4366596, Applied Biosystems™). Real-time PCR was performed using TaqMan™ Universal Master Mix II, with UNG (cat. 4440038, Applied Biosystems™), and the relative abundance of miRNA was normalized to RNU6B. All procedures were processed according to the manufacturer’s protocols. The mixture of small RNA-specific stem-looped RT Primer, small RNA-specific forward and reverse PCR primer, and small RNA-specific TaqMan™ MGB probe was obtained from Applied Biosystems™. The following assays were used: has-miR-29a (Assay ID. 002112), has-miR-29b (Assay ID. 000413), and RNU6B (Assay ID. 001093).

### Immunoprecipitation

Cells were lysed using Pierce™ IP Lysis Buffer (cat. 87787, Thermo Scientific™, USA) supplemented with a protease inhibitor. The lysates were quantified using the BCA assay to ensure a precise distribution of 1 mg per sample. They were incubated at 4 °C with rotation, along with a 1:100 (recommended dilution) MED12 antibody (#14360) and 20 µL of Protein A/G PLUS-Agarose (cat. sc-2003, Santa Cruz, USA). Subsequently, the washing step was performed four times with 1 mL of Pierce™ IP lysis buffer, and the samples were boiled at 100 °C, adding 2× SDS PAGE buffer in a heat block. The samples were then analyzed by Western blotting.

### Fluorescence imaging

The H3122/MED12 KO cell line, transfected with the GFP-tagging mutant MED12 plasmid, was seeded in a 60 mm cell culture dish. Fixed positions were imaged using the ZOE Fluorescent Cell Imager (Bio-Rad, California, USA) in the Green Channel (Excitation: 480/17 nm, Emission: 517/23 nm) to capture fluorescence images.

### Animal experiments

Animal experiment procedures were approved by the Institutional Animal Care and Use Committee (IACUC-2022-12-246) of the Asan Medical Center (AMC) and Animal Research: Reporting In Vivo Experiments (ARRIVE) guidelines. Five-week-old BALB/c Slc-nu/nu male mice were purchased from Japan SLC Inc (Shizuoka Laboratory Center, Japan). After 1 week, the H3122 and H3122/MED12 KO xenograft model was established upon subcutaneous injection of 5 × 10^6^ cells/100 µL PBS into the right hind flanks. When the average tumor volume reached approximately 100 mm3, the mice were randomized into three groups (vehicle, ceritinib, trametinib; *n* = 3 mice per group). Ceritinib (25 mg/kg) and trametinib (1 mg/kg) were administered by oral gavage once per day for up to 3 weeks (21 days). The size of the tumors was assessed every other day by caliper measurement, and the lesion volumes were calculated using the following formula: 0.5 × L × W^2 (mm^3), in which L refers to the length, and W refers to the width of each tumor. P-values and percentage of tumor growth inhibition ratio (T/C) values were calculated at the end of the experiment. Animal procedures were approved by the Asan Medical Centre Institutional Animal Care and Use Committee and Animal Research: Reporting In Vivo Experiments (ARRIVE) guidelines.

### Immunohistochemistry

Tumor tissues extracted from the xenograft mouse model were fixed in formalin for approximately 1–2 weeks. The tumor tissues were then processed to create paraffin blocks, and sections were mounted on microscope slides. Hematoxylin and eosin (H&E) staining, as well as immunohistochemistry (IHC) staining for MED12 and Ki-67, were performed, and we received technical support for these procedures from the Comparative Pathology Core facility at the ConveRgence mEDIcine research canTer (CREDIT), Asan Medical Center.

### AACR GENIE BPC NSCLC v2.0-public dataset analysis

The GENIE BPC NSCLC v2.0-public dataset includes clinical information for 1,846 NSCLC patients provided by four institutions: MSKCC, DFCI, VICC, and UHN [40, 41]. The dataset was analyzed through cBioPortal platform. Initially, to identify patients with EGFR-TKI and ALKi sensitive mutations, we obtained a Patient ID/Sample ID list with mutations classified as ‘biomarkers predictive of response to a drug’ from standard of OncoKB for EGFR and ALK. We then excluded those not profiled for the presence of MED12 mutations from this list. Next, from the cohort identified in the previous step, we selected patients/samples with a history of EGFR-TKI and ALKi treatment. The selected samples were acquired before the treatment, not after. Finally, the last cohort consisted of patients with both EGFR-TKI or ALKi sensitive mutations and a treatment history. In this cohort a comprehensive data analysis, including genomic alteration event frequency, oncoprint, mutation frequency, and progression-free survival-imaging (PFS-I), was carried out.

### TCGA PanCancer atlas and Gene Expression Omnibus (GEO) dataset analysis

The TCGA PanCancer Atlas dataset was accessed through the cBioPortal platform. This dataset included clinical information for lung adenocarcinoma patients, who were categorized into subgroups, considering the entire population (*n* = 505) and those (*n* = 202) with a documented treatment history. Subsequently, Survival-related data, including overall survival (OS) and progression-free survival (PFS), were retrieved and processed based on the presence or absence of MED12 mutation.

The Gene Expression Omnibus (GEO) dataset was accessed using the Kaplan-Meier Plotter platform. Specifically, the GSE3141 (*n* = 111), GSE29013 (*n* = 55), and GSE68465 (*n* = 462) datasets were utilized, and these datasets encompass clinical information for lung cancer patients, providing a foundation for gene expression-based survival prediction analyses. Probe ID 211342_x_at and 203506_s_at were selected for MED12, and the associated expression levels were extracted. Survival-related data, specifically overall survival (OS) and first progression survival (FP), were retrieved and processed based on MED12 expression levels.

### Gene Set Enrichment Analysis (GSEA) from The Cancer Genome Atlas (TCGA) database and MED12 knock-out cell lines

GSEA was conducted using the GSEA v4.3.2 software from the Broad Institute. Gene sets from the Molecular Signatures Database (Human MSigDB v2023.2.Hs, updated October 2023) were utilized, focusing on the KEGG_LEGACY subset of curated gene sets. For this study, RNA-seq data were obtained from The Cancer Genome Atlas (TCGA) Lung Adenocarcinoma (LUAD) cohort via the Genomic Data Commons (GDC) Data Portal (https://www.cancer.gov/tcga). Additionally, total mRNA was extracted from MED12 knock-out H3122 and PC9 cell lines, as well as their respective parental cell lines, using the easy-spin™ Total RNA Extraction Kit (cat. 17221, iNtRON Biotechnology). RNA-seq libraries were prepared using the TruSeq Stranded mRNA Library Prep Kit (Illumina). Library preparation and sequencing were outsourced to MACROGEN CO., where the samples were sequenced on an Illumina platform to generate high-quality RNA-seq data.

### Olink proteomics analysis

Proteomic profiling of culture media samples from MED12 knock-out H3122 and PC9 cell lines, as well as their respective parental cell lines, was performed using the Olink Proteomics platform with the Olink Target 96 Inflammation panel. The assays were conducted by Olink Proteomics (Uppsala, Sweden) following the manufacturer’s instructions. Protein expression levels were reported using Normalized Protein eXpression (NPX) values, a relative quantification unit on a log2 scale. Data normalization and log2 transformation were performed. Comparative analysis among groups was conducted using NPX values, and statistical tests were performed accordingly. Bar plots visualized protein fold changes.

### Extraction and analysis of chromatin-bound and soluble cellular fractions

Chromatin-bound and soluble cellular fractions were extracted from MED12 knock-out H3122 and PC9 cell lines, as well as their respective parental cell lines, using the Subcellular Protein Fractionation Kit for Cultured Cells (cat. 78840, Thermo Scientific™) according to the manufacturer’s instructions. Cells were lysed and centrifuged to separate the soluble fraction (supernatant) from the chromatin-bound fraction (pellet). Proteins from both fractions were quantified using the BCA Protein Assay Kit (Thermo Scientific™) and analyzed by Western blotting to assess the distribution and levels of target proteins.

### Trans-well insert co-culture assay

To investigate the effects of cytokines released from MED12 knock-out cell lines, a Trans-well insert co-culture assay was performed. MED12 knock-out H3122 and PC9 cell lines, as well as their respective parental cell lines, were seeded in the upper inserts of a Trans-well system (cat. 37006, SPL Life Sciences, 0.4 μm pore size). Parental cell lines were cultured in the bottom wells of a 6-well plate. This setup allowed cytokines secreted by the cells in the upper inserts to diffuse and interact with the cells in the bottom wells.

For the clonogenic assay, cells in the bottom wells were treated with RTKi every 3–4 days for 2 weeks, followed by crystal violet staining to assess colony formation. Additionally, after the 48-hour co-culture, cells were treated with or without RTKi for 3 h. The cells were then collected for western blotting. This design allowed us to compare the effects of cytokine exposure and RTKi treatment on MED12 knock-out and parental cell lines.

### Growth factor array

To investigate the potential influence of growth factors on the AKT and ERK1/2 pathways, we employed the Human Growth Factor Array C1 from RayBiotech (cat. AAH-GF-1-4). Culture media from MED12 knock-out cell lines (H3122 and PC9), as well as their respective parental cell lines, were collected after 48 h of incubation. The media were centrifuged to remove cell debris, and the supernatant was used for analysis. The array membranes were blocked and then incubated overnight with the prepared culture media at 4 °C. Following washes, biotin-conjugated antibodies specific to the growth factors were added, and the membranes were further incubated with HRP-conjugated streptavidin. Detection was performed using the SuperSignal™ West Femto Maximum Sensitivity Substrate (cat. 34095). Relative expression levels of growth factors were compared between MED12 knock-out and parental cell lines, normalized to internal positive controls on the array. This analysis provided comprehensive profiling of growth factors in the culture media, offering insights into their role in influencing the AKT and ERK1/2 pathways.

## Results

### MED12 mutation induces RTK inhibitor resistance and predicts poor survival in NSCLC patients

**Fig. 1:**
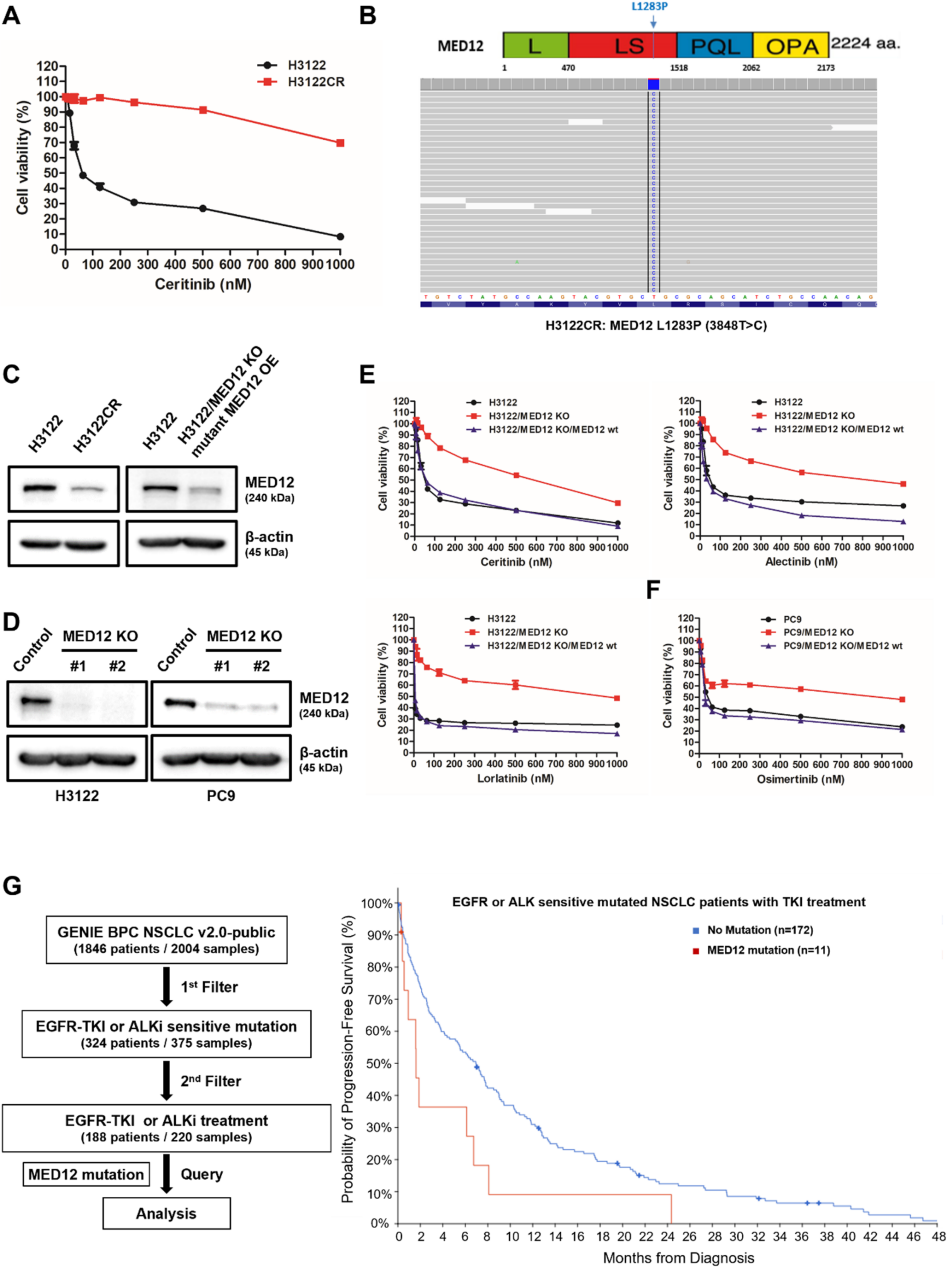
MED12 mutation induces resistance to RTK inhibitors in NSCLC via the suppression of its expression and predicts poor survival to RTK inhibitors in NSCLC patients **A** Cell viability of the ceritinib-resistant H3122 cell line (H3122CR) was assessed using the MTS assay. **B** Targeted sequencing analysis of H3122CR identified the presence of the L1283P (3848T > C) mutation in the MED12 gene, represented as a proportion (%) among various gene mutations. **C** Western blotting analysis confirmed decreased MED12 expression in both ceritinib-resistant and mutant MED12 overexpression cell line compared to the control group. **D** Western blot analysis validated the efficient knockout of MED12 in the generated knockout (KO) cell lines, exhibiting a notable reduction in MED12 protein expression compared to the control group. **E**,** F** MTS assay was performed to evaluate the sensitivity of MED12 KO cell lines to RTK inhibitors (ceritinib, alectinib, lorlatinib, and osimertinib). Cell viability of MED12 KO cell lines was compared to the respective control cell lines. Re-expression of wild-type MED12 in KO cells restored RTK inhibitor sensitivity to levels comparable to the parental cells. **G** Kaplan-Meier survival analysis of progression-free survival (PFS) for EGFR-TKI or ALKi treated NSCLC patients with MED12 mutations compared to those without. The hazard ratio (HR) was 1.979 (p-value = 0.0246), indicating an unfavorable prognosis for patients with MED12 mutations following RTKi treatment

We identified a novel marker of ceritinib resistance in EML4-ALK rearranged NSCLC by establishing a ceritinib-resistant H3122 cell line (H3122CR) through gradual ceritinib exposure. Resistance was confirmed via MTS assay (Fig. 1A). Whole-exome sequencing (WES) of H3122CR revealed the presence of an L1283P (3848T > C) mutation in the MED12 gene (Fig. 1B). Correspondingly, MED12 expression was significantly reduced in the resistant H3122CR cells (Fig. 1C). To explore whether loss of MED12 function contributes to RTK inhibitor resistance, we utilized CRISPR/Cas9-mediated knockout of MED12 in both the H3122 (ALKi-sensitive) and PC9 (EGFR-TKI-sensitive) cell lines, which was confirmed by assessing MED12 expression (Fig. 1D). MED12 knockout (KO) cell lines exhibited resistance to various RTK inhibitors (ceritinib, alectinib, lorlatinib, and osimertinib) as demonstrated by MTS assays, and restoring wild-type MED12 fully reinstated sensitivity to these inhibitors, reaching levels comparable to the parental cells (Fig. 1E, F).

To investigate the response of patients with MED12 mutations to RTK inhibitors, we utilized the GENIE BPC NSCLC v2.0 public database from the AACR Project GENIE, accessed through cBioPortal, to select an appropriate patient cohort [40, 41]. Initially, we identified 324 patients with known mutations sensitive to EGFR-TKIs or ALK inhibitors (ALKi) from a total of 1846 NSCLC patients. Among these, we selected 188 patients who had been treated with EGFR-TKIs or ALKis. Subsequent analysis was conducted to assess the presence or absence of MED12 mutations within this cohort (Supplementary Fig. S1A). In this group, 155 patients (84%) exhibited mutations sensitive to EGFR-TKIs, 32 patients (18%) had mutations sensitive to ALKis, and 11 patients (6%) had MED12 mutations (Supplementary Fig. S1B). Specifically, among EGFR-TKI-sensitive patients, 10 (6.45%) carried MED12 mutations, and among ALKi-sensitive patients, 1 (3.13%) harbored a MED12 mutation (Supplementary Fig. S1C). Analysis of progression-free survival (PFS) revealed a hazard ratio (HR) of 1.979 (*p* = 0.0246) for patients with MED12 mutations, indicating a poorer prognosis for those receiving RTK inhibitors, including both EGFR-TKIs and ALKis (Fig. 1H).

### The L1283P (3848T > C) mutation in the MED12 gene induces its proteasomal degradation, leading to disruption of the MED12 complex

**Fig. 2:**
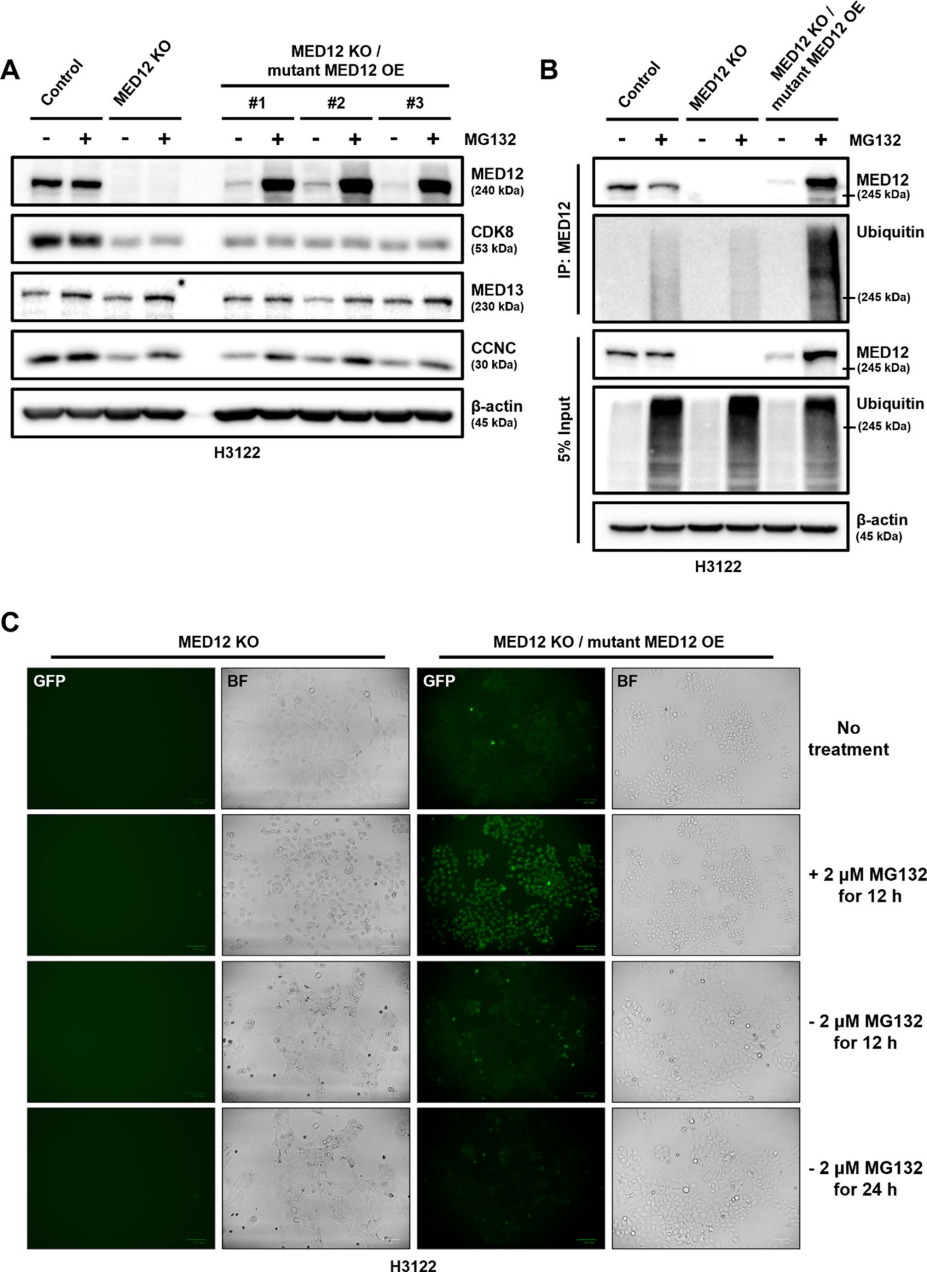
MED12 mutation, L1283P, induces its proteasomal degradation, which lead to break of MED12 complex. **A** Western blot analysis showing the protein expression levels of MED12 complex components (MED12, CDK8, MED13, CCNC) in the parental cell line (H3122) and MED12 knockout (KO) cell line with mutant MED12 overexpression (OE) before and after treatment with 2 µM MG132 (proteasome inhibitor) for 24 h. **B** Co-immunoprecipitation was performed to assess the direct interaction between the mutant MED12 and ubiquitin. Protein lysates from the MED12 KO/mutant MED12 OE cell line were immunoprecipitated with anti-MED12 antibody, followed by immunoblotting with anti-ubiquitin antibody. **C** Fluorescence imaging of GFP-tagged MED12 in the MED12 KO/mutant MED12 OE cell line before and after treatment with MG132, demonstrating the blockade of ubiquitin-mediated proteasomal degradation

In a previous study, we identified the L1283P mutation in the MED12 gene and observed a decrease in MED12 expression in the ceritinib-resistant H3122CR cell line (Fig. 1B, C). To investigate the underlying mechanism of this phenomenon, we transfected a MED12 vector harboring the 3848 T > C mutation into a MED12 knockout (KO) cell line. We then treated the cells with MG132, a proteasome inhibitor, to examine the effect of proteasomal degradation on mutant MED12 expression. In the parental H3122 cell line, no significant change in MED12 expression was observed following MG132 treatment. However, in the MED12 KO cell line with overexpression of the mutant MED12 (OE), a marked increase in MED12 expression was observed upon MG132 treatment (Fig. 2A).

To further explore the degradation mechanism, we performed co-immunoprecipitation experiments, which demonstrated that the mutant MED12 directly binds to ubiquitin and undergoes ubiquitination in the presence of MG132 treatment (Fig. 2B). Additionally, we confirmed the inhibition of ubiquitin-mediated proteasomal degradation of GFP-tagged MED12 by fluorescence imaging (Fig. 2C). Notably, we observed that MED12 degradation led to a reduction in the expression of CDK8 and CCNC, key components of the MED12 complex, suggesting that the degradation of MED12 disrupts the integrity of the Mediator complex.

### Inflammatory cytokine release induced by MED12 mutation drives RTK inhibitor resistance via MEK/ERK pathway activation, but Not the PI3K/AKT pathway

**Fig. 3:**
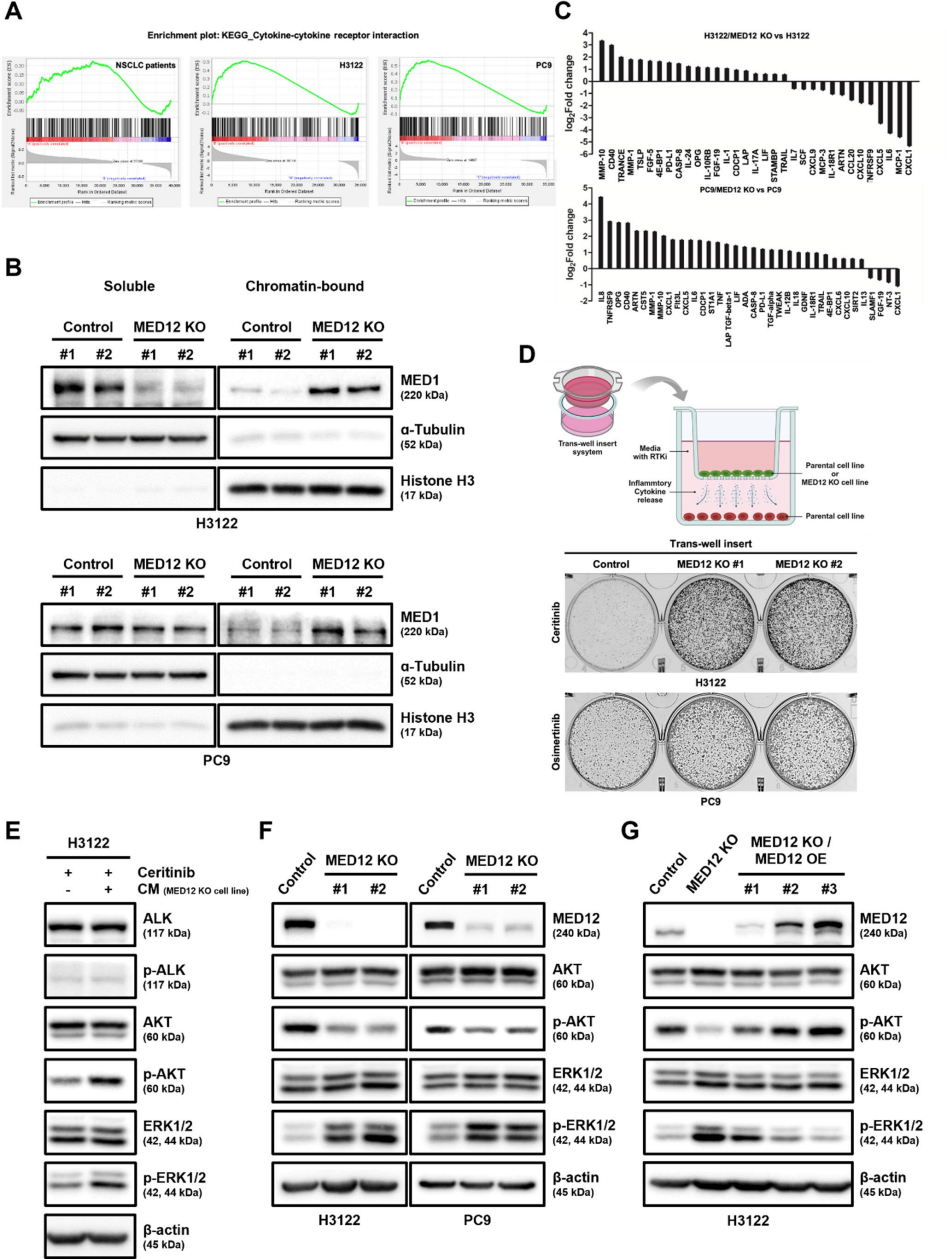
Inflammatory cytokines release by MED12 mutation induce RTK inhibitor resistance through only the MEK/ERK pathway activation, not the PI3K/AKT pathway. **A** Gene Set Enrichment Analysis (GSEA) of RNA-seq data from NSCLC patients with MED12 mutations, as well as MED12 knock-out H3122 and PC9 cell lines, showing significant enrichment in the cytokine-cytokine receptor interaction gene set of the KEGG_LEGACY subset. **B** Western blot analysis confirming elevated chromatin-bound MED1 levels in MED12 knock-out H3122 and PC9 cell lines compared to parental cell lines. **C** Olink proteomics analysis of culture media from MED12 knock-out cell lines, identifying increased levels of inflammatory cytokines. **D** Cell image of the trans-well insert co-culture system used to expose parental NSCLC cell lines to cytokines released from MED12 knock-out cell lines, resulting in increased RTKi resistance, assessed by clonogenic assay. Schematic of the trans-well co-culture system was created with BioRender.com. **E** Western blot analysis showing the activation of both AKT and ERK1/2 pathways in parental cell lines exposed to cytokines from MED12 knock-out cell lines. **F** In H3122/MED12 KO cell lines, inhibition of the AKT pathway and activation of the ERK1/2 pathway were observed by Western blotting analysis. **G** Western blot analysis demonstrating the reactivation of the AKT pathway and suppression of the ERK1/2 pathway in H3122/MED12 KO cell lines after overexpression of wild type MED12

To investigate the mechanism of RTK inhibitor (RTKi) resistance induced by the functional loss of MED12, we analyzed RNA sequencing (RNA-seq) data from non-small cell lung cancer (NSCLC) patients with MED12 mutations available in The Cancer Genome Atlas (TCGA) database, as well as from MED12 knockout (KO) H3122 and PC9 cell lines, utilizing Gene Set Enrichment Analysis (GSEA). Remarkably, all three datasets revealed significant enrichment in gene sets related to cytokine signaling (Fig. 3A and Supplementary Fig. S2). Previous studies have demonstrated that MED12 deficiency in CAR-T cells enhances inflammatory cytokine production and increases the core Mediator complex’s transcriptional activity [42]. Consistent with these findings, we observed that the chromatin-bound MED1, a key component of the Mediator complex associated with active transcription, was elevated in MED12 KO cell lines compared to their parental counterparts (Fig. 3B). To confirm the release of inflammatory cytokines at the protein level, we performed Olink proteomics analysis on the culture media of MED12 KO cell lines. This analysis revealed a significant increase in various inflammatory factors (Fig. 3C). To investigate whether these cytokines contribute to RTKi resistance, we established a system using trans-well inserts to continuously expose parental cell lines to cytokines released from MED12 KO cell lines (Fig. 3D). This exposure resulted in a noticeable increase in RTKi resistance in the parental cell lines (Fig. 3D), accompanied by the activation of both the AKT and ERK1/2 signaling pathways (Fig. 3E). We further explored whether growth factors, which are a subclass of cytokines, might influence the activation of the AKT or ERK1/2 pathways. Using a growth factor array that analyzed 33 distinct factors, we examined the culture media and found no significant changes in most growth factors. However, the MED12 KO H3122 cell lines exhibited elevated levels of PDGF-AA, PDGF-BB, and VEGF-A (Supplementary Fig. S3A). Despite these changes, Western blot analysis of RTK receptors known to be activated by these growth factors, such as PDGFRβ and VEGFR2, revealed no activation of these receptors (Supplementary Fig. S3B). These results suggest that the inflammatory cytokines, rather than the growth factors, are specifically responsible for activating the AKT and ERK1/2 pathways. Interestingly, contrary to previous studies [43–49], we observed that only the ERK1/2 pathway, and not the AKT pathway, was activated in MED12 KO NSCLC cell lines exposed to the same cytokines (Fig. 3F). To directly assess the role of MED12 expression, we modulated its levels and observed corresponding changes in the activation of both the AKT and ERK1/2 pathways, which notably reverted to the parental pattern upon restoration of wild-type MED12 expression. (Fig. 3G). Furthermore, ceritinib sensitivity was proportionally recovered as the expression level of wild-type MED12 gradually increased (Supplementary Fig. S4).

These findings indicate that the release of inflammatory cytokines due to MED12 functional loss is a sufficient mechanism for inducing RTKi resistance in NSCLC. Furthermore, while both the AKT and ERK1/2 pathways can be activated by inflammatory cytokines, MED12 KO NSCLC cell lines specifically exhibit activation of the ERK1/2 pathway, highlighting a unique and distinct mechanism of RTKi resistance.

### MEK Inhibitor, Trametinib, Overcomes RTK Inhibitor Resistance Induced by MED12 Mutation

**Fig. 4:**
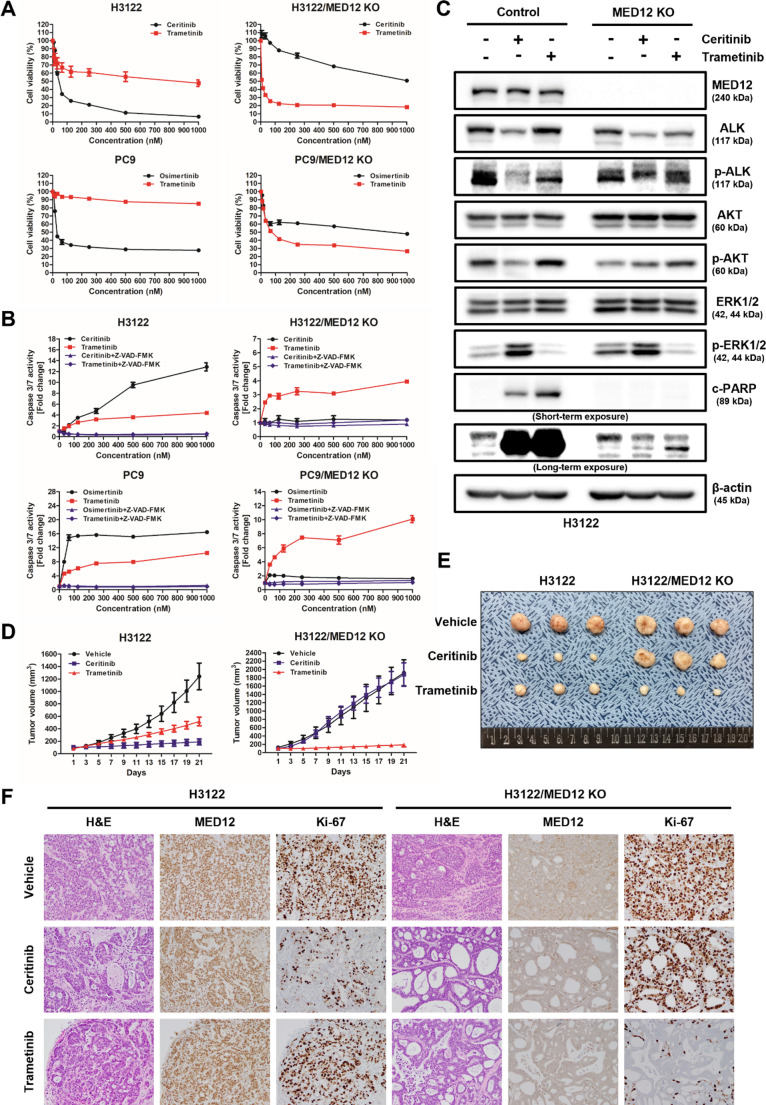
MEK inhibitor, trametinib, alone overcomes RTK-induced resistance by repression of MED12 expression by its mutation. **A** MTS assay was performed to assess the cell viability of MED12 KO cell lines and control cell lines treated with ceritinib/osimertinib and trametinib. **B** Caspase 3/7 assay was performed to evaluate apoptosis in MED12 KO cell lines and control cell lines treated with ceritinib, osimertinib, or trametinib, with or without co-treatment of the pan-caspase inhibitor Z-VAD-FMK. **C** Western blot analysis showing the expression of the apoptosis marker cleaved PARP in MED12 KO cell lines treated with trametinib compared to control groups. Ceritinib and trametinib were each administered at a concentration of 200 nM for 72 h. **D** Tumor volume measurements of xenograft mouse models implanted with parental cell line (H3122, *n* = 3) and MED12 knockout (KO) NSCLC cells (H3122/MED12 KO, *n* = 3) and treated with daily administration of vehicle, ceritinib, or trametinib for three weeks. **E** Representative images of tumors obtained from the xenograft mouse model showing the differences in tumor growth among the treatment groups. **F** Immunohistochemistry (IHC) analysis performed on tumor tissues derived from the xenograft mouse model to evaluate the expression levels of Ki-67, a proliferation marker, and MED12

The results from MTS assays demonstrated that the MEK inhibitor, trametinib, effectively inhibited the growth of two types of RTK inhibitor-resistant MED12 knockout (KO) cell lines (ceritinib- and osimertinib-resistant) (Fig. 4A). Consistently, caspase 3/7 activity assays showed significantly increased apoptotic activity in trametinib-treated MED12 KO cells compared to control cells, further supporting the enhanced apoptotic sensitivity upon MED12 functional loss (Fig. 4B). Additionally, Western blot analysis revealed a strong expression of cleaved PARP, a marker of apoptosis, in the trametinib-treated RTK inhibitor-resistant MED12 KO cell lines, further indicating its pro-apoptotic effect (Fig. 4C).

To further evaluate the therapeutic potential of trametinib in overcoming RTK inhibitor resistance in the context of MED12 functional loss, we used a xenograft mouse model (Supplementary Fig. S5). Parental (H3122) and MED12 KO NSCLC cells (H3122/MED12 KO) were implanted into BALB/c nude (immunodeficient) mice to allow tumor growth. After tumor establishment, the mice were treated with either vehicle, ceritinib, or trametinib for three weeks, and tumor growth was monitored. In this model, the growth of tumors derived from H3122/MED12 KO cells was significantly suppressed in the trametinib-treated group compared to both the vehicle and ceritinib-treated groups, suggesting that trametinib is effective in inhibiting tumor progression despite the presence of MED12 functional loss (Fig. 4D, E).

Immunohistochemistry (IHC) analysis was performed on tumor tissues collected from the xenograft model to assess the expression of the proliferation marker Ki-67. The results showed a significant reduction in Ki-67 expression in the trametinib-treated MED12 KO tumors compared to those treated with ceritinib, indicating that trametinib effectively inhibits tumor cell proliferation (Fig. 4F).

These findings highlight trametinib as a promising therapeutic option for the treatment of RTK inhibitor-resistant NSCLC associated with MED12 functional loss. Trametinib’s ability to inhibit tumor growth in vivo, particularly in the context of MED12 mutations, suggests its potential as a strategy to overcome resistance to RTK inhibitors. Further investigations are needed to elucidate the underlying mechanisms and optimize treatment protocols for this combination therapy.

### Inhibition of MED12-YAP interaction leads to increased PTEN expression and suppression of the AKT pathway

**Fig. 5:**
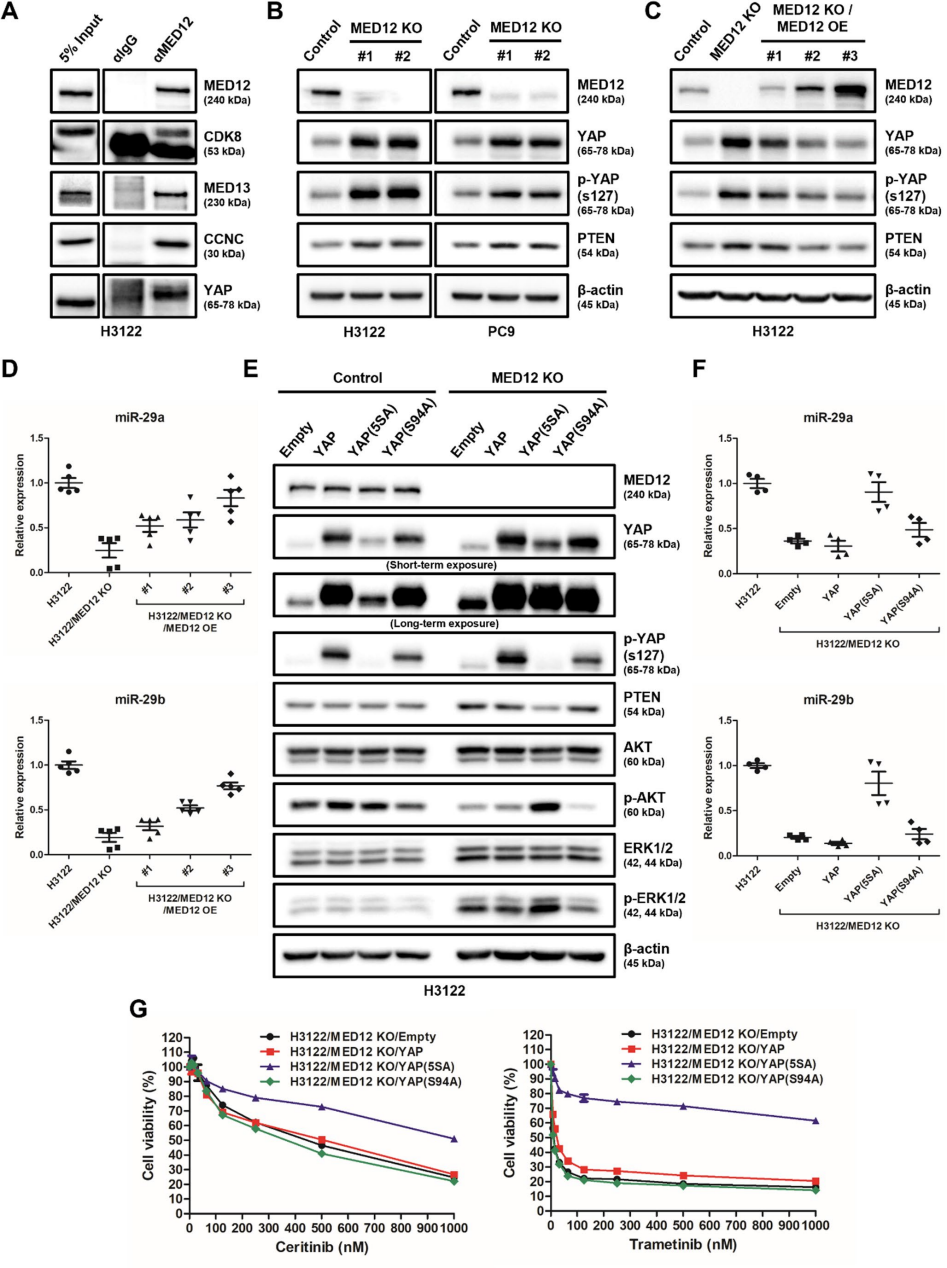
Inhibiting physical interaction between MED12 and YAP leads to increased PTEN expression and subsequent inhibition of the AKT pathway. **A** Co-immunoprecipitation was performed to confirm the direct interaction between MED12 and YAP. Protein lysates from the cell lines were immunoprecipitated with anti-MED12 antibody, followed by immunoblotting with anti-YAP antibody. **B** Western blot analysis showing increased levels of phospho-YAP (ser127) and PTEN in the MED12 KO cell line compared to the control, indicating the inhibition of the PI3K/AKT pathway. **C** Restoration of wild type MED12 expression in the MED12 KO cell line resulted in decreased levels of phospho-YAP (ser127) and PTEN, indicating the reactivation of the PI3K/AKT pathway. **D** The expression levels of miR-29, a mediator of PTEN suppression by YAP, were downregulated in the MED12 KO cell line and restored after wild-type MED12 recovery, as determined by qPCR analysis. **E** Western blot analysis showing the effects of YAP overexpression (YAP, YAP-5SA, YAP-S94A) in both the parental (H3122) and MED12 KO (H3122/MED12 KO) cell lines. Changes in PTEN and phospho-AKT (p-AKT) levels were evaluated. **F** The expression levels of miR-29 in the different YAP overexpressed cell lines were evaluated by qRT-PCR. **G** Resistance to ceritinib and trametinib in the various YAP overexpressed cell lines was assessed using MTS assay

Previous research has shown that MED12 directly interacts with YAP [50], a regulator of the PI3K/AKT signaling pathway [51]. To further investigate the relationship between MED12 and the AKT pathway, we performed co-immunoprecipitation assays to confirm the direct interaction between MED12 and YAP (Fig. 5A).

In the MED12 knockout (KO) cell line, we observed an increase in phosphorylated YAP (Ser127, which is inactive) and PTEN, a known negative regulator of the PI3K/AKT pathway (Fig. 5B). To verify whether MED12 regulates these effects, we reintroduced wild-type MED12 into the MED12 KO cell line, which resulted in a decrease in both phospho-YAP (Ser127) and PTEN levels (Fig. 5C). Additionally, we examined miR-29 expression, a microRNA that suppresses PTEN via YAP [52], and found that miR-29 expression was decreased in the MED12 KO cell line but was restored upon re-expression of wild-type MED12 (Fig. 5D).

To further explore the role of YAP in mediating the effects of MED12 functional loss on the AKT pathway, we transfected empty vector, YAP, YAP (5SA, an overactive form), and YAP (S94A, an inactive form) vectors into both parental (H3122) and MED12 KO (H3122/MED12 KO) cell lines [39]. In the parental cell line, overexpression of YAP variants did not lead to significant changes in PTEN or p-AKT levels. However, in the MED12 KO cell line, overexpression of YAP or YAP (S94A) did not affect PTEN or p-AKT, whereas overexpression of YAP (5SA) resulted in significant reductions in PTEN expression and increases in phospho-AKT levels (Fig. 5E). Additionally, only YAP (5SA)-overexpressing cells showed a significant increase in miR-29 expression (Fig. 5F) and exhibited resistance to ceritinib and trametinib (Fig. 5G), indicating that YAP (5SA) overexpression confers resistance to RTK inhibitors. To validate the regulatory role of YAP on PTEN expression in the context of MED12 loss, we overexpressed wild-type YAP, YAP (5SA), and YAP (S94A) in the MED12 KO cell line. We found that YAP (5SA) overexpression resulted in a significant decrease in PTEN expression (Fig. 5E) and an increase in miR-29 levels (Fig. 5F) compared to wild-type or YAP (S94A)-overexpressing MED12 KO cells. Moreover, in the H3122 parental cell line, both YAP wild-type and YAP (5SA) overexpression induced resistance to ceritinib, while only YAP (5SA) overexpression significantly enhanced resistance to ceritinib and trametinib in the H3122/MED12 KO cell line (Fig. 5G).

These findings suggest that the inhibition of the physical interaction between MED12 and YAP increases PTEN expression and suppresses the AKT pathway. Additionally, YAP overactivation (specifically in its 5SA form) induces PTEN suppression and activation of the AKT pathway, leading to enhanced resistance to RTK inhibitors such as ceritinib and trametinib. This highlights the importance of the MED12-YAP interaction in regulating PTEN expression and AKT pathway activation, as well as its potential role in resistance to targeted therapies in NSCLC.

## Discussion

In non-small cell lung cancer (NSCLC), targeted therapies based on RTK mutations have become clinically prevalent, but drug resistance remains a significant challenge, and research to address this issue is ongoing. To identify new biomarkers and mechanisms associated with RTKi resistance in EML4-ALK rearranged NSCLC and to develop treatment strategies, we established resistance cell lines against the second-generation ALK inhibitor, ceritinib. Through targeted sequencing, we discovered a mutation L1283P (3848T > C) in the MED12 gene (Fig. 1B), and confirmed that this mutation leads to a reduction in MED12 expression in ceritinib-resistant cells (Fig. 1 C).

According to previous reports, reduced MED12 expression can lead to TKI resistance in EML4-ALK rearranged NSCLC cell lines (H3122) and EGFR-TKI sensitive cell lines through the regulation of TGF-beta receptor signaling [32]. Therefore, we performed MED12 knockout (KO) in H3122 (ALK inhibitor sensitive) and PC9 (EGFR-TKI sensitive) cell lines (Fig. 1D), which resulted in decreased MED12 expression and confirmed resistance to RTKi in both cell lines (Fig. 1E, F). Additionally, we transfected the MED12 mutation (L1283P) into MED12 KO cell lines (H3122/MED12 KO) and confirmed MED12 expression. Compared to H3122 cells, MED12 was not expressed in H3122/MED12 KO/mutant MED12 cells. However, after treatment with the proteasome inhibitor MG132, a significant amount of mutant MED12 increased in H3122/MED12 KO/mutant MED12 cells (Fig. 2 A). This indicates that the proteasome recognizes the MED12 mutation (L1283P) protein as misfolded and degrades it (Fig. 2B, C). This suggests that reduced MED12 expression in NSCLC patients with the MED12 mutation can lead to the loss of its function. Interestingly, the expression levels of MED12 complex components CDK8, MED13, and CCNC were also low in H3122/MED12 KO and H3122/MED12 KO/mutant MED12 cells, indicating a decreased function of the MED12 complex.

Previous studies have reported that reduced MED12 expression can lead to unfavorable outcomes in chemotherapy treatment through the regulation of TGF-beta receptor signaling [32]. However, there are limitations in explaining MED12 mutations and RTKi-specific responses in actual patients. To demonstrate a direct clinical correlation of this issue, we selected a meaningful patient cohort for our research purpose from the GENIE BPC NSCLC v2.0-public database provided by the AACR Project GENIE on cBioPortal (Supplementary Fig. S1A, B; Table 1). We confirmed that the presence of MED12 mutations among patients treated with EGFR-TKI or ALKi may actually indicate poor prognosis (Fig. 1G). Furthermore, we additionally confirmed that the presence of MED12 mutations was associated with poor clinical outcomes in the overall patient cohort (Supplementary Fig. S6, S7). These findings indicate a need for further investigation of MED12 as a potential biomarker, independent of RTKi-sensitive mutation status.

**Table 1:** Patient and cancer characteristics for those with both EGFR-TKI or ALKi sensitive mutations and a treatment history

	No mutation	MED12 mutation
Patient characteristic	*n* = 177, n (%)	*n* =11, n (%)
Age at diagnosis		
Range	26-84	48-78
Sex		
Female	121 (68.4)	7 (63.6)
Male	56 (31.6)	4 (36.4)
Race		
White	116 (65.5)	9 (81.8)
Chinese	30 (16.9)	1 (9.1)
Black	12 (6.8)	1 (9.1)
Unknown	9 (5.1)	–
Other Asian	5 (2.8)	–
Other	3 (1.7)	–
American Indian, Aleutian, or Eskimo	2 (1.1)	–
Ethnicity		
Non-Spanish; non-Hispanic	170 (96.0)	10 (90.9)
Mexican (includes Chicano)	3 (1.7)	1 (9.1)
Spanish surname only	2 (1.1)	–
Spanish NOS or Hispanic NOS or Latino NOS	1 (0.6)	–
Unknown whether Spanish or not	1 (0.6)	–
Smoking status		
Never smoker	100 (56.5)	6 (54.5)
Former smoker (quit >1 year)	59 (33.3)	4 (36.4)
Former smoker (quit <1 year)	12 (6.8)	1 (9.1)
Current smoker	4 (2.3)	–
Former smoker (unknown time)	2 (1.1)	–
Histology		
Adenocarcinoma	149 (84.2)	9 (81.8)
Carcinoma	14 (7.9)	2 (18.2)
Squamous cell	5 (2.8)	–
NA	5 (2.8)	–
Other histologies/mixed tumor	3 (1.7)	–
Small cell carcinoma	1 (0.6)	–
Stage at diagnosis		
Stage IV	119 (67.2)	9 (81.8)
Stage III	26 (14.7)	2 (18.2)
Stage I	16 (9.0)	–
Stage II	16 (9.0)	–
Institution		
MSK	137 (77.4)	8 (72.7)
DFCI	27 (15.3)	2 (18.2)
VICC	13 (7.3)	1 (9.1)

*Abbreviation*: *NOS* not otherwise specified

To investigate the mechanism by which the MED12 mutation induces RTKi resistance, we performed Gene Set Enrichment Analysis (GSEA) based on TCGA data from NSCLC patients with EGFR mutations or ALK rearrangements and compared clinical data according to the presence of MED12 mutations. This analysis revealed a significant increase in cytokine-related gene sets in patient cohorts with MED12 mutations compared to those without MED12 mutations. Interestingly, a similar pattern was observed in RNA-seq data from two MED12 knockout NSCLC cell lines (Fig. 3A). These results are consistent with previous studies showing an increase in cytokine-related gene sets in MED12-deficient T cells [42].

Previous studies have shown that the MED1-containing mediator module, when bound to the MED12 complex’s kinase module, inhibits the role of transcription factors. When separated from the MED12 complex, MED1 is known to act as a transcription factor involved in cytokine expression by binding to RNA polymerase II (Pol II) [42]. To confirm the mechanism of cytokine expression mediated by MED12, we separated chromatin-bound and soluble cellular fractions and observed that chromatin-bound MED1 was relatively increased compared to soluble MED1 in MED12 knockout NSCLC cell lines (Fig. 3B). These findings suggest that the reduced MED12 expression caused by the mutation may disrupt the MED12 complex, leading to the separation of the mediator module containing MED1. The separated mediator module then binds to Pol II, thereby promoting cytokine gene transcription.

To verify whether the cytokine-related gene sets from GSEA analysis led to actual increases in cytokine release, we used Olink proteomics to assess the release of inflammatory cytokines in the conditioned medium (CM) of two MED12 knockout cell lines (Fig. 3C). We further demonstrated that CM containing cytokines from these MED12 KO cell lines can induce RTKi resistance in RTKi-sensitive cells (H3122, PC9) and as already known [43–49], cytokines can reactivate the PI3K/AKT and MEK/ERK pathways (Fig. 3D, E). These results suggest that, in addition to the well-known RTK resistance mechanisms such as “new mutations within the RTK domain (On target)” and “activation of other RTKs (Off target)”, inflammatory cytokines may play a role in inducing RTKi resistance through a new mechanism.

In MED12 KO cell lines, we expected that inflammatory cytokines would activate both the PI3K/AKT and MEK/ERK pathways. However, we found that the PI3K/AKT pathway was actually inactive (Fig. 3F, G). Considering that both the PI3K/AKT and MEK/ERK pathways are key downstream signaling pathways directly related to cell proliferation and survival [10, 53], this suggests that the MED12 KO NSCLC cell lines are highly dependent on the MEK/ERK pathway since AKT is already inhibited. Based on this finding, to establish a clinically applicable treatment strategy for RTKi-resistant NSCLC induced by MED12 mutations, we tested whether the MEK inhibitor trametinib could be effective as a single agent in NSCLC with MED12 mutations resistant to RTK inhibitors. We confirmed that the two MED12 KO cell lines were resistant to ceritinib and osimertinib, but trametinib exhibited strong anti-cancer effects in both cell lines (Fig. 4A). To verify whether this effect was due to the reduced expression of MED12, we restored wild-type MED12 expression in MED12 KO NSCLC cell lines and confirmed the recovery of sensitivity to RTK inhibitors (Fig. 1E, F and Supplementary Fig. S4). In addition, we observed significantly increased apoptotic activity in trametinib-treated MED12 KO cells, as evidenced by both caspase 3/7 activity assays and the expression of cleaved PARP (Fig. 4B, C). These findings further support the notion that MED12 loss enhances the apoptotic response to MEK inhibition.

To elucidate the mechanism by which the MED12 mutation inhibits the PI3K/AKT pathway, we focused on YAP, a transcriptional co-regulator. YAP is known to physically interact with the MED12 complex (Fig. 5A) [50] and binds to the miR-29 promoter to induce its expression. The expressed miR-29 then directly binds to the RNA of PTEN, inhibiting PTEN expression. Since PTEN is a well-known inhibitor of PI3K/AKT pathway activation, YAP can affect the PI3K/AKT signaling pathway through PTEN. When YAP is phosphorylated, it cannot bind to the miR-29 promoter, leading to the expression of PTEN, which in turn results in the inhibition of PI3K/AKT activity.

We confirmed the direct interaction between MED12 and YAP through immunoprecipitation (IP) analysis (Fig. 5A). We observed increased phosphorylation of YAP ser127 (inactive form) and increased PTEN expression in MED12 KO cell lines (Fig. 5B). These results indicate that reduced MED12 expression leads to the loss of function of the MED12 complex, causing the phosphorylation and subsequent inactivation of YAP, which in turn results in increased PTEN expression. For further validation, we restored wild-type MED12 in MED12 KO cells and observed a decrease in YAP ser127 phosphorylation and PTEN expression (Fig. 5C). Additionally, miR-29a/b expression was suppressed in MED12 KO cells, and restoration of wild-type MED12 led to the recovery of miR-29a/b expression (Fig. 5D).

To confirm the direct impact of YAP on the regulation of PTEN and AKT, we established stable cell lines in H3122 and H3122/MED12 KO cells using empty vector, YAP, YAP-5SA (overactive), and YAP-S94A (inactive) vectors. In H3122 cells, none of the four YAP vectors caused significant changes in PTEN, p-AKT, or p-ERK1/2 levels, suggesting that MED12 and YAP are well-associated. In MED12 KO H3122 cells, despite the overexpression of YAP and YAP(S94A), there were no significant differences in PTEN and p-AKT levels compared to the empty vector, indicating that reduced MED12 expression impairs the normal function of YAP. Only YAP-5SA, the hyperactive form of YAP, was able to forcibly activate YAP and restore PTEN and p-AKT levels to resemble those of the parent cell line (Fig. 5E). The miR-29a/b regulated by YAP was only restored in YAP-5SA overexpressing H3122/MED12 KO cells (Fig. 5F). Furthermore, the observed increase in ceritinib resistance in YAP-5SA overexpressing H3122/MED12 KO cells aligns with previous studies indicating that YAP induces drug resistance [52, 54–58], and this resistance to trametinib is due to the reactivation of p-AKT (Fig. 5G).

These results demonstrate that MED12 plays a role in maintaining the normal transcription factor function of YAP. Additionally, our findings help explain previous research showing that CDK8 directly phosphorylates YAP [59] and that direct inhibition of CDK8 enhances the therapeutic efficacy of MEK inhibitors [60].

We also conducted xenograft animal experiments to evaluate the effect of trametinib on MED12 mutation-induced RTKi resistance. Our results showed that the MEK inhibitor trametinib effectively reduced tumors based on H3122/MED12 KO cells (Fig. 4D). These findings suggest that trametinib could be the optimal drug for treating RTK inhibitor resistance induced by MED12 mutations.

**Fig. 6:**
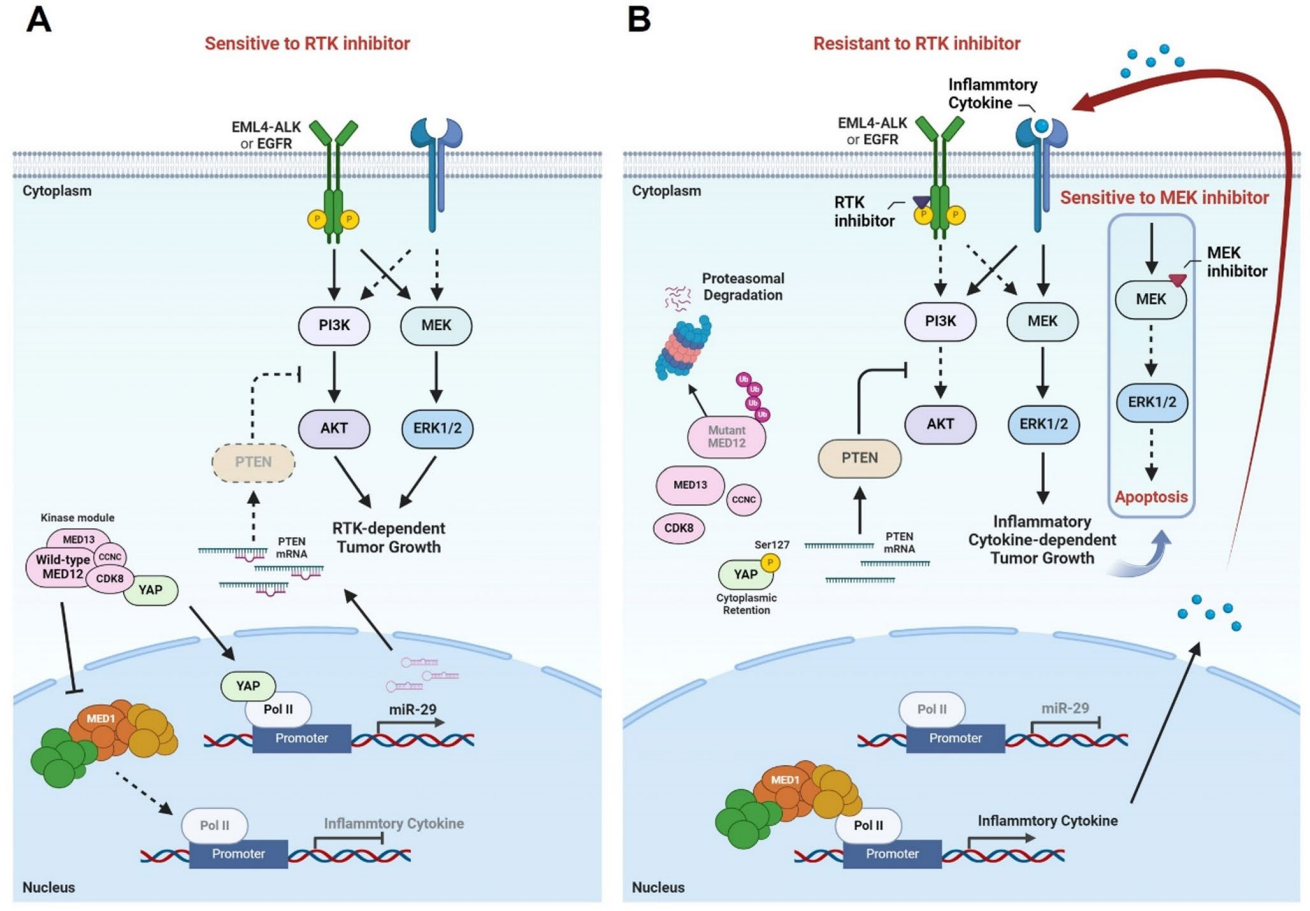
MEK inhibitor could be the most suitable treatment option for MED12 mutation-induced RTK inhibitor-resistant NSCLC. Created with BioRender.com Schemas illustrating the identified mechanisms of MED12 mutation-mediated resistance to RTK inhibitions. **A** Targeting EGFR or ALK in EGFR-mutated or EML4-ALK/wild-type MED12 cells. **B** Although MED12 mutation-induced inflammatory cytokine induces both PI3K/AKT activation and MEK/ERK activation, MED12 mutation blocks PI3K/AKT activation by PTEN induction via YAP regulation. Therefore, MEK inhibitor alone provides sufficient benefit for MED12-mutated RTKi-resistant NSCLC. Solid lines indicate the effects

Generally, a well-known resistance mechanism to RTK inhibitors is the reactivation of other RTKs. Therefore, in clinical practice, drugs targeting reactivated RTKs are used, or if such targeted therapies are unavailable, combination therapies that inhibit both the PI3K/AKT and MEK/ERK pathways, common downstream pathways of RTKs, are applied. However, these combination therapies are limited due to increased drug toxicity. Previous studies have described that inhibition of MED12 suppresses the physical interaction between MED12 and TGF-βR2, leading to TGF-βR2 activation [32]. However, our research suggests that patients with RTK inhibitor resistance due to MED12 mutations can achieve sufficient therapeutic efficacy with a MEK inhibitor alone, rather than relying on unnecessary combination therapies. This finding is expected to provide new treatment guidelines for NSCLC patients (Fig. 6).

To support this model, we further investigated the molecular mechanism linking MED12 to AKT signaling. A recent study has shown that YAP and MYC cooperate to suppress PTEN expression, and that MED12 loss may modulate this regulatory axis [61]. Consistent with these findings, we observed significantly reduced c-MYC protein levels in MED12 knockout cells (data not shown), accompanied by increased PTEN expression and suppressed AKT activity. Although further investigation is required to clarify the direct regulatory relationship between MED12 and MYC, our results suggest that functional loss of MED12 attenuates AKT-mediated survival signaling, thereby increasing the vulnerability of cancer cells to MEK inhibition.

Together, these data not only provide a mechanistic rationale for the use of trametinib monotherapy in MED12-mutant NSCLC but also offer a biologically coherent explanation for how RTKi resistance in this subset of patients can be managed without resorting to toxic combination therapies.

Our study proposes two new perspectives. First, we anticipate direct clinical applicability. Despite the reporting of multiple drug resistances in patients with MED12 inhibition [31, 62], MED12 has been recognized as an important biomarker [33, 63]. However, research in this area has been stagnant and insufficient over the past decade. Our study highlights the utility of MED12 as a new companion diagnostic marker for identifying RTKi-resistant patients and suggests the potential expansion of indications for the MEK inhibitor trametinib for the treatment of these patients. Second, the MED12-CDK8-YAP-PTEN-PI3K/AKT mechanism discovered in this study is expected to contribute to future mechanistic research in other studies. Recent reports indicate that CDK8 inhibition enhances the anti-cancer effects of MEK inhibitors [60], and in EGFR mutant lung cancer, the combination of EGFR inhibitors with YAP/TEAD inhibitors has shown synergistic effects [58]. These studies suggest a correlation with CDK8 and YAP, which aligns with our findings. Additionally, recent reports emphasizing the relationship between MED12 and immunology show favorable outcomes with immune checkpoint inhibitors in patients with MED12 mutations [64]. Furthermore, genetically engineered CAR-T cells with MED12 KO exhibit increased anti-tumor activity [42]. These observations suggest that MED12 might regulate PD-L1 expression through MED12-YAP interactions, highlighting the importance of MED12 in immunotherapy.

In conclusion, this study found that inflammatory cytokine release induced by MED12 mutations leads to RTK inhibitor resistance, and that this resistance can be effectively overcome with MEK inhibitors alone.

## Supplementary Information

Below is the link to the electronic supplementary material.

Supplementary file1 (DOCX 2765 kb)

## Data Availability

All data needed to evaluate the conclusions in this article are available in the methods and/or the supplementary material of this article. Any additional information on the findings of this study is available on request from the corresponding author.
