# Supplementary material for: MED12 mutation induces RTK inhibitor resistance in NSCLC via MEK/ERK pathway activation by inflammatory cytokines
Source: Cell Mol Life Sci. 2025 Aug 20;82(1):314. doi: 10.1007/s00018-025-05791-w (PMC12367601; doi:10.1007/s00018-025-05791-w)
Supplement: Supplementary file 1 — Supplementary file1 (DOCX 2765 kb) [file 18_2025_5791_MOESM1_ESM.docx]

**Supplementary Figures**


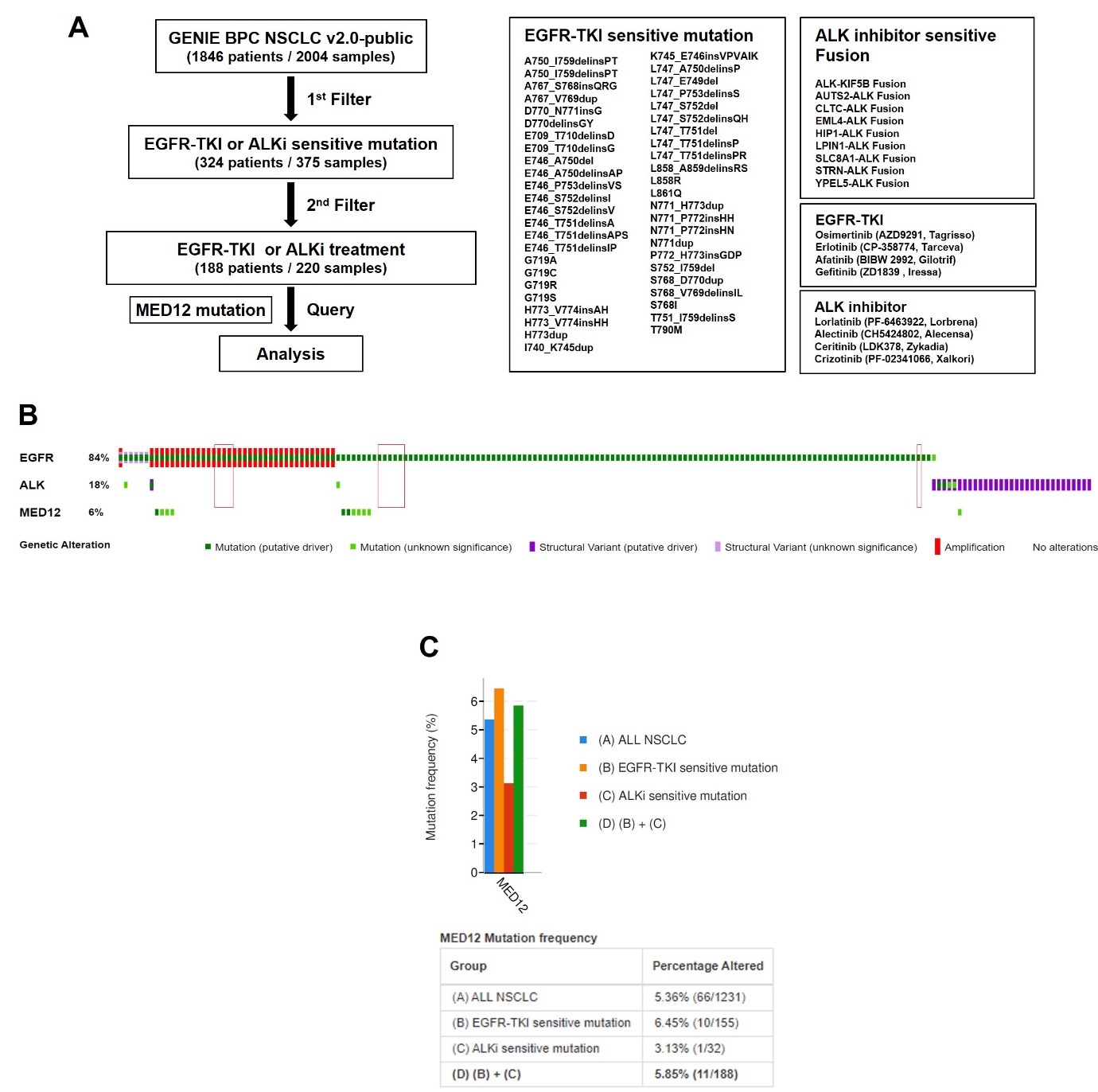


**Supplementary Figure 1. The MED12-mutant NSCLC patients harboring RTK, such as ALK or EGFR, sensitive mutations show poor prognosis for RTK inhibitors.**

**(A)** Patient cohort selection process using the GENIE BPC NSCLC v2.0-public database. **(B)** Oncoprint showing distribution of patients in the selected cohort based on EGFR, ALK and MED12 mutation status. **(C)** MED12 mutation frequency within both EGFR-TKI and ALKi sensitive mutation groups.


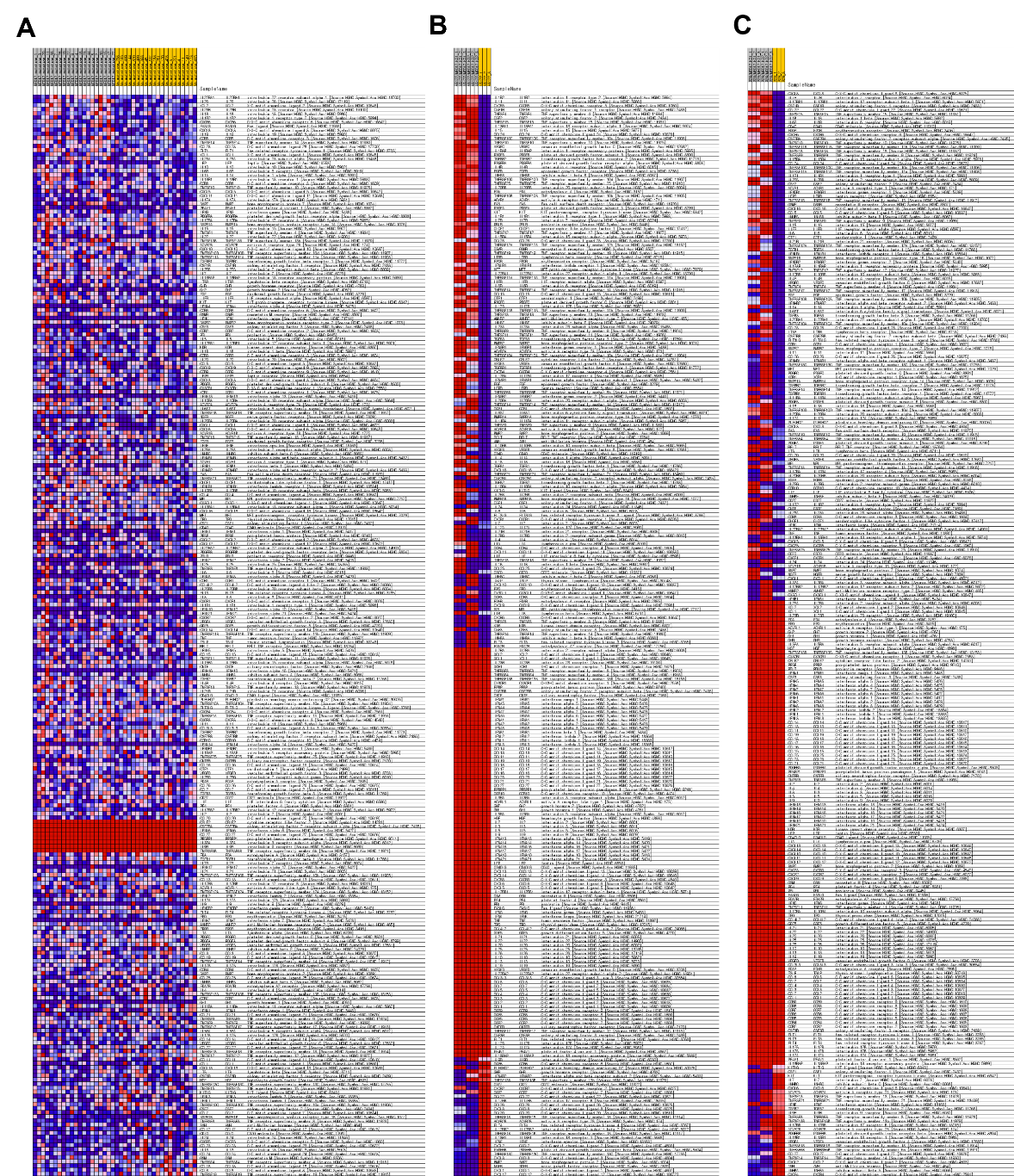


**Supplementary Figure 2. Heat map analysis of inflammatory gene associated with MED12 mutation.**

(A) Heat map showing enrichment of inflammatory response-related genes in non-small cell lung cancer (NSCLC) patients harboring MED12 mutations from The Cancer Genome Atlas (TCGA) dataset.
(B, C) Heat map of inflammatory gene expression changes in MED12 knockout (B) H3122 and (C) PC9 cells, highlighting consistent upregulation of cytokine signaling pathways across both cell lines. Gene expression data were analyzed using GSEA (Gene Set Enrichment Analysis), and expression levels are represented by color gradients (red: upregulated, blue: downregulated).


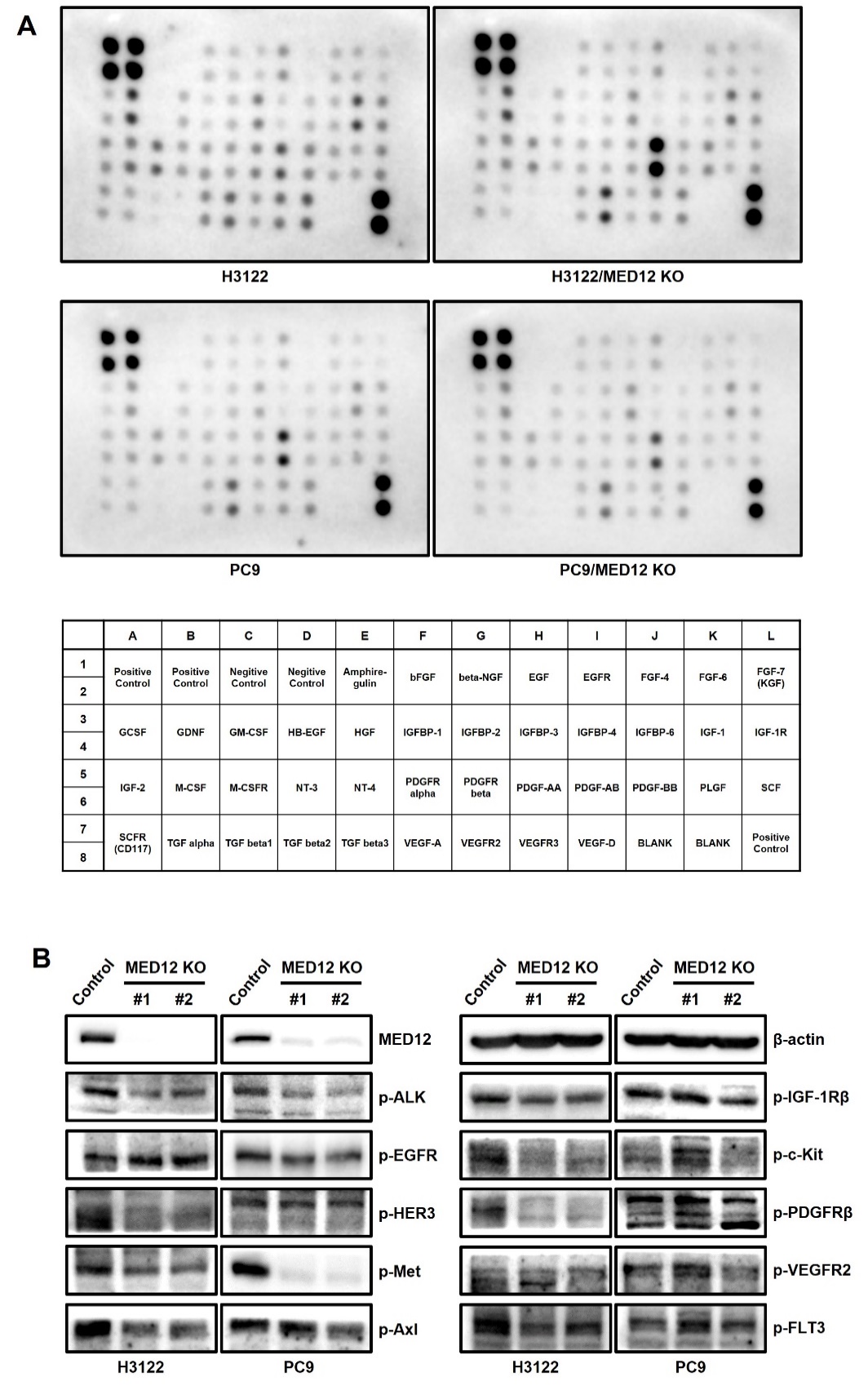


**Supplementary Figure 3. Analysis of growth factors and RTK receptors in MED12 knock-out cell lines.**

**(A)** Human Growth Factor Array Result. Culture media from MED12 knock-out cells (H3122 and PC9 cell lines), as well as their respective parental cell lines, were collected after 48 hours of incubation. **(B)** Western blot analysis for RTK activation in the parental (H3122 and PC9) and MED12 KO (H3122/MED12 KO and PC9/MED12 KO) cell lines.


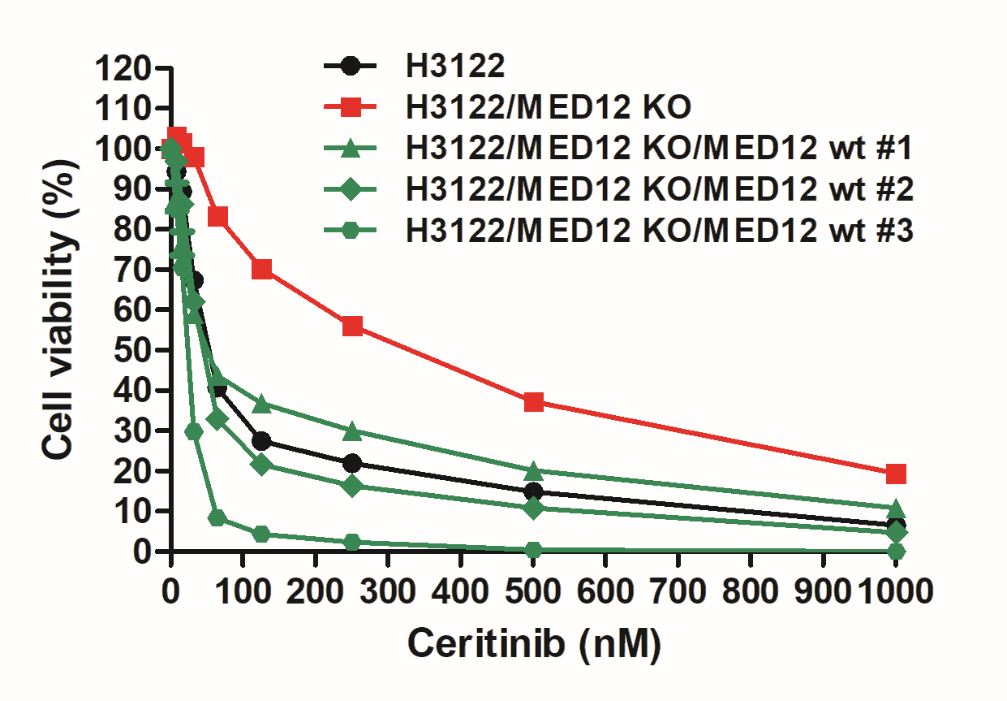


**Supplementary Figure 4. MED12 expression-dependent enhancement of ceritinib sensitivity in MED12 KO cells.**

Ceritinib sensitivity in MED12 knockout (KO) H3122 cells increased in proportion to the level of wild-type MED12 expression. At higher expression levels, sensitivity approached or exceeded that of the parental cells. Cell viability was assessed using MTS assays.


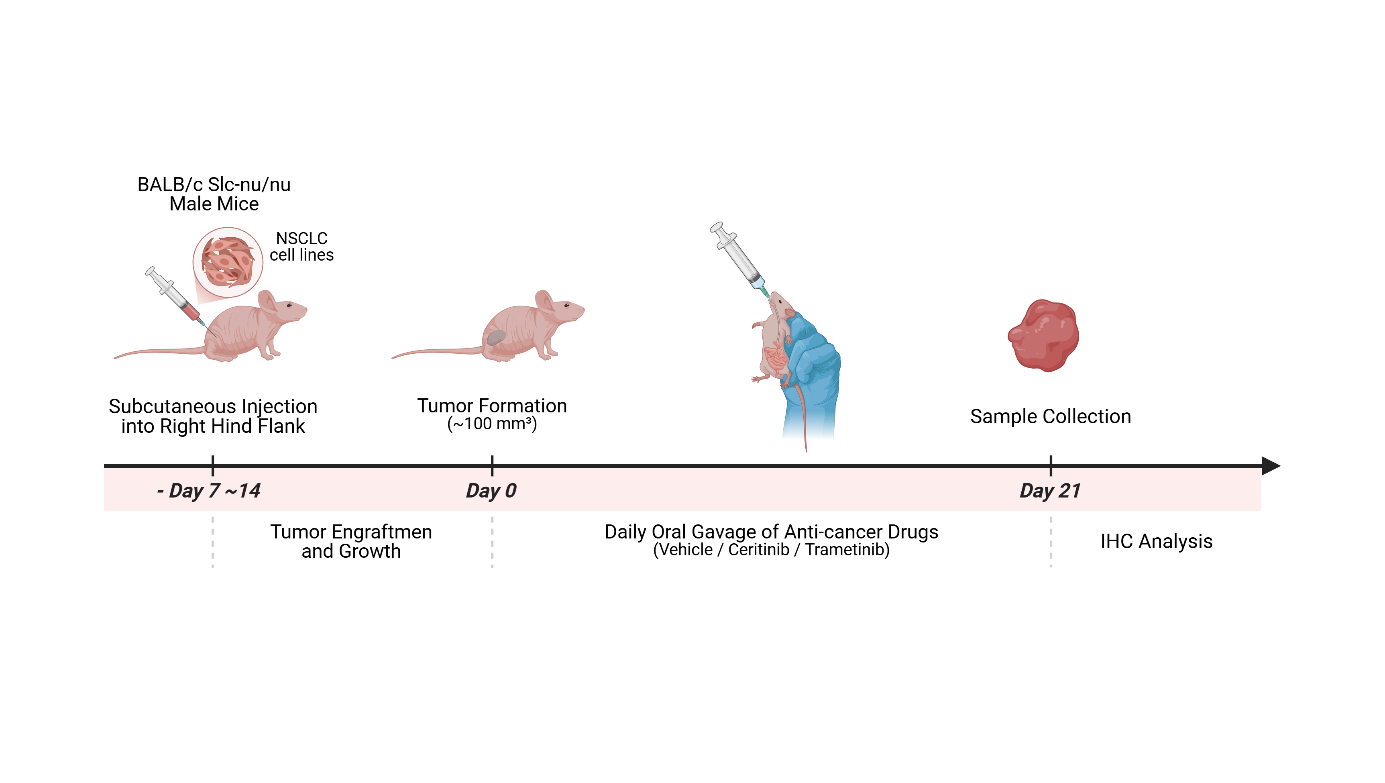


**Supplementary Figure 5. Experimental timeline and treatment scheme of xenograft mouse model administered with ceritinib and trametinib. Created with BioRender.com.**

NSCLC cells were subcutaneously injected into the right flank of immunodeficient mice. After 7–14 days, when the tumor volume reached approximately 100 mm³, mice were randomly grouped and orally treated with ceritinib or trametinib daily for 21 days. Tumor sizes were monitored regularly, and tumors were harvested at the end of treatment for IHC analysis.


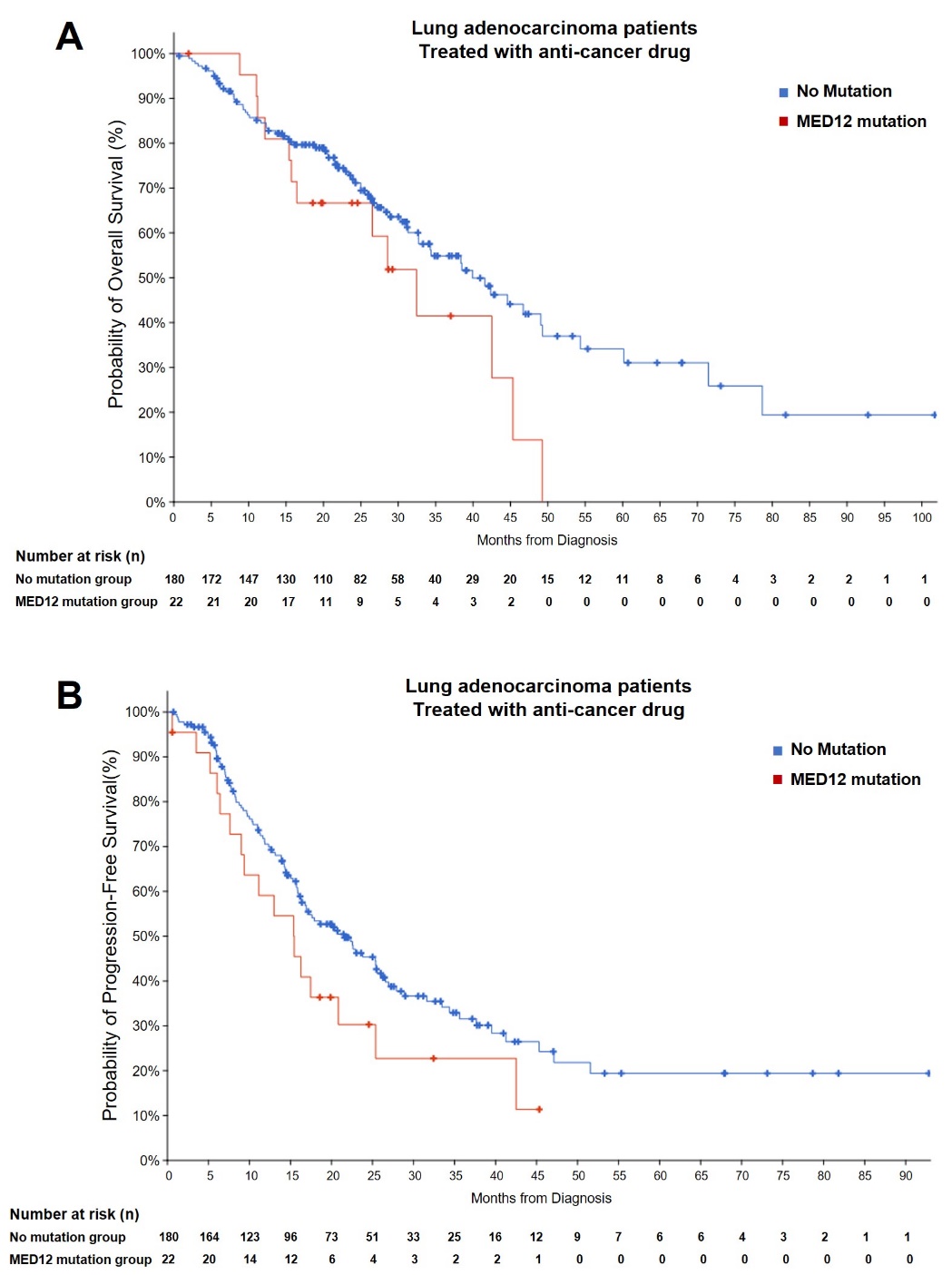


**Supplementary Figure 6. Analysis of survival endpoints in the lung adenocarcinoma patients from TCGA PanCancer Atlas dataset.**

**(A)** Overall survival according to the presence or absence of MED12 mutation. (Left: No

filtered patients, HR=1.643, p-value=0.0268; Right: anti-cancer drug treated patients,

HR=1.621, p-value=0.103) **(B)** Progression-free survival according to the presence or absence of MED12 mutation. (Left: No filtered patients, HR=1.502, p-value=0.0589; Right: anti-cancer drug treated patients, HR=1.521, p-value=0.106)


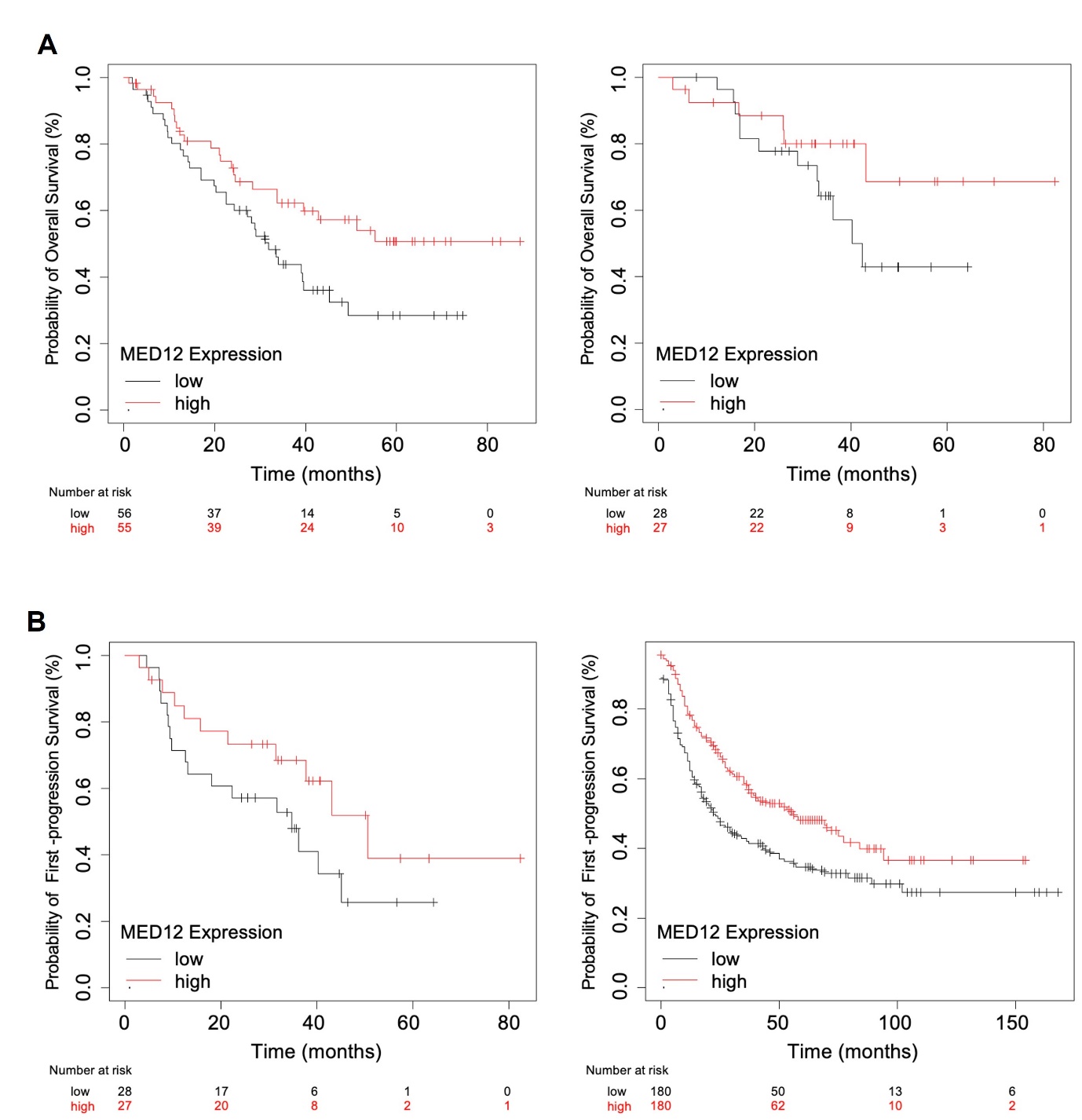


**Supplementary Figure 7. Analysis of survival endpoints in the NSCLC patients from Gene Expression Omnibus (GEO) dataset.**

**(A)** Overall survival according to the MED12 mRNA expression level. (Left: GSE3141 dataset,

Probe ID=211342_x_at, HR=1.786, p-value=0.031; Right: GSE29013 dataset, Probe ID=203506_s_at, HR=1.961, p-value=0.17) **(B)** First-progression survival according the MED12 mRNA expression level. (Left: GSE29013 dataset, Probe ID=203506_s_at, HR=1.754, p-value=0.15; Right: GSE68465, Probe ID=211342_x_at, HR=1.538, p-value=0.002
